# A novel recyclable organocatalyst for the gram-scale enantioselective synthesis of (*S*)-baclofen

**DOI:** 10.3762/bjoc.19.133

**Published:** 2023-11-24

**Authors:** Gyula Dargó, Dóra Erdélyi, Balázs Molnár, Péter Kisszékelyi, Zsófia Garádi, József Kupai

**Affiliations:** 1 Department of Organic Chemistry and Technology, Budapest University of Technology and Economics, Műegyetem rkp. 3., H-1111 Budapest, Hungary; 2 Department of Pharmacognosy, Semmelweis University, Üllői út. 26, H-1085 Budapest, Hungary

**Keywords:** baclofen, catalyst recovery, lipophilic cinchona squaramide, organocatalysis, stereoselective catalysis

## Abstract

Synthesizing organocatalysts is often a long and cost-intensive process, therefore, the recovery and reuse of the catalysts are particularly important to establish sustainable organocatalytic transformations. In this work, we demonstrate the synthesis, application, and recycling of a new lipophilic cinchona squaramide organocatalyst. The synthesized lipophilic organocatalyst was applied in Michael additions. The catalyst was utilized to promote the Michael addition of cyclohexyl Meldrum’s acid to 4-chloro-*trans*-β-nitrostyrene (quantitative yield, up to 96% ee). Moreover, 1 mol % of the catalyst was feasible to conduct the gram-scale preparation of baclofen precursor (89% yield, 96% ee). Finally, thanks to the lipophilic character of the catalyst, it was easily recycled after the reaction by replacing the non-polar reaction solvent with a polar solvent, acetonitrile, with 91–100% efficiency, and the catalyst was reused in five reaction cycles without the loss of activity and selectivity.

## Introduction

In today’s chemical industry, catalytic processes are of paramount importance. In particular, the application of asymmetric organocatalysts is receiving increased attention [1–4]. This is illustrated by the fact that in 2021 the Nobel Prize in Chemistry was awarded for the discovery of asymmetric organocatalysis [5]. The use of organocatalysts has been a major breakthrough in the realization of enantioselective transformations. Stereoselective synthesis is essential in the pharmaceutical industry, as the development of drugs often requires the production of enantiomerically pure chiral compounds [6–8].

The application of organocatalysts is well-established in several organic reactions, including but not limited to aldol reactions [6], Michael additions [9], Mannich reactions [10], aza-Henry reactions [11], and Diels–Alder cycloadditions [12–13]. Although the benefits of organocatalysts are undoubted, their synthesis is often a long and expensive process. Therefore, for sustainable application, the cost-efficient recovery and reuse of organocatalysts are critical issues. Fortunately, a wide range of recycling options are known in the literature, often based on liquid–solid phase separation [14].

Catalyst recycling can be achieved, for example, by immobilizing the catalysts to a solid support [15], e.g., silica gel [16–18], organic polymers [19–21], magnetic nanoparticles [22–23], or by membrane separation, e.g., using organic solvent nanofiltration (OSN) [24–26], which methods can be easily implemented in flow systems. Accordingly, the main recycling methods rely on the immobilization of catalysts on heterogeneous supports, however, this could often lead to the deterioration of activity and/or selectivity [27]. A possible solution to avoid these drawbacks is the heterogenization of the catalyst after a homogeneous reaction.

For example, by incorporating a lipophilic side chain [28] on the organocatalyst that does not interfere with its catalytic activity thanks to a linker between the catalyst and lipophilic units. In this way, a significant difference in polarity can be achieved between the catalyst and the other components of the reaction mixture. The lipophilic *O*-alkylated gallic acid unit increases the solubility of the organocatalyst in less polar solvents, such as DCM or toluene but leads to the precipitation of the organocatalyst in polar solvents, including MeOH or MeCN. As a result, the recycling of the organocatalysts can be achieved in a simple step by centrifugation or filtration.

**Figure 1:**
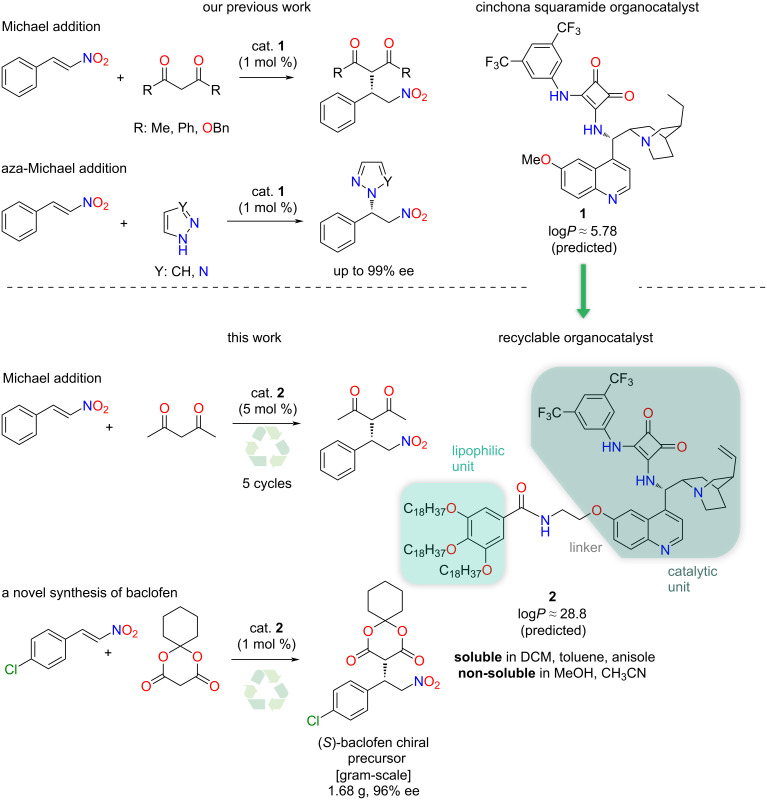
Application of cinchona squaramide **1** and recyclable, lipophilic cinchona squaramide organocatalysts **2** in a new, gram-scale stereoselective synthesis of baclofen precursor.

Previously, we have demonstrated the homogeneous and heterogeneous recycling of cinchona-based organocatalysts [20,25–26,29]. Continuing our work, we aimed to synthesize a novel, recyclable lipophilic cinchona squaramide organocatalyst. Its catalytic activity and recyclability were examined in a new stereoselective synthesis of baclofen, which is used to treat muscle spasms [30]. Finally, the catalyst was easily recycled by centrifugation over five reaction cycles without significant loss of activity (Figure 1).

## Results and Discussion

### Synthesis of the lipophilic cinchona squaramide organocatalyst

Previously, we successfully applied quinine-derived squaramide (SQ) organocatalyst **1** in stereoselective Michael and aza-Michael additions with excellent enantiomeric excess values [26]. Our aim was to recycle this catalyst easily by incorporating a lipophilic unit, which leads to a drastic increase (5.78 to 28.8) in the log*P* value of the organocatalyst **2**. The recyclable organocatalyst can be divided into three units: the catalytic unit, the linker, and the lipophilic tag with octadecyl chains (Figure 1).

**Scheme 1:**
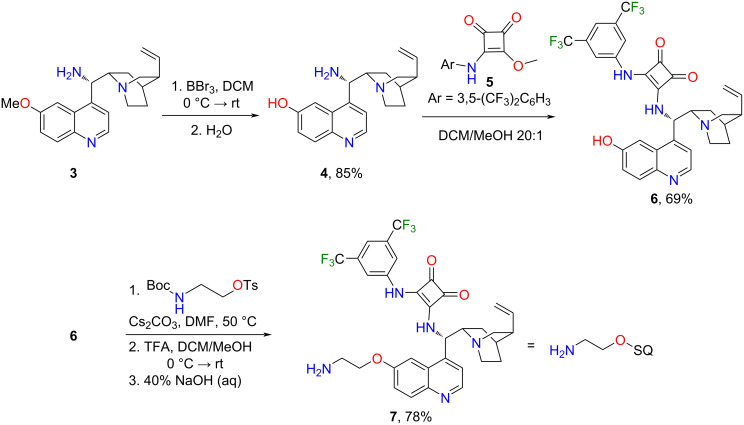
Synthesis of demethylated cinchona squaramide organocatalyst and the incorporation of the flexible 2-aminoethylene linker.

The cinchona amine **3** was prepared starting from the naturally occurring quinine [31]. The gained catalyst was demethylated using BBr_3_ to give alcohol **4**. The demethylated cinchona amine was reacted with half-squaramide [9] **5**, resulting in demethylated squaramide **6**. A short and flexible linker was applied between the catalytic and lipophilic units to avoid a decrease in the catalytic activity. The demethylated cinchona squaramide **6** was reacted with *O*-*p*-toluenesulfonyl-*N*-Boc-ethanolamine. The protecting group was removed using trifluoroacetic acid, followed by a neutralization step, gaining the cinchona squaramide organocatalyst **7** with a linker (Scheme 1).

**Scheme 2:**
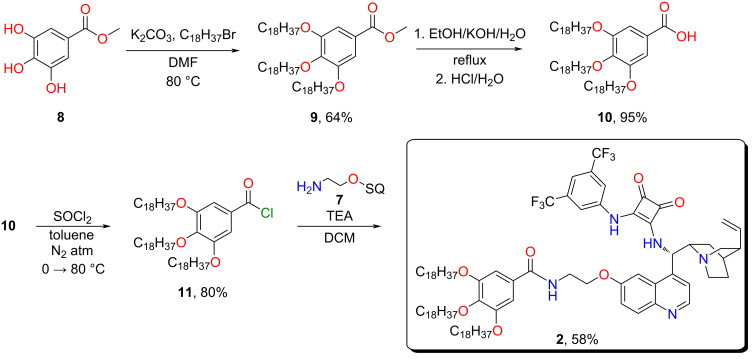
Synthesis of the lipophilic tag from methyl gallate (**8**) and attachment to the cinchona squaramide.

The lipophilic unit from methyl gallate (**8**) was gained by following a literature procedure [32] with minor modifications. The octadecyl groups were attached to the hydroxy groups using Williamson-type ether synthesis. The octadecylated gallic acid ester **9** was hydrolyzed under basic conditions in an ethanol/water mixture. After the reaction, the pH of the mixture was adjusted to 4 with hydrochloric acid, which resulted in the precipitation of the product **10** in excellent yield (95%). Next, carboxylic acid **10** was converted into the corresponding acyl chloride **11** with thionyl chloride. Finally, the cinchona squaramide with linker **7** and the octadecylated gallic acid chloride **11** were coupled to form an amide using triethylamine as a base. The crude product was purified by chromatography and precipitated from acetonitrile to gain the lipophilic organocatalyst **2** (Scheme 2).

### Application and recycling of the lipophilic cinchona-squaramide organocatalyst in the stereoselective Michael addition

To prove that the previously applied catalytic unit kept its activity, the lipophilic organocatalyst **2** was applied in the stereoselective Michael addition of *trans*-β-nitrostyrene (**12**) and acetylacetone (**13**). Choosing the best solvent for the reaction is crucial, thus, solubility tests were carried out (Table 1). Since homogeneous catalysts usually exhibit higher activity and selectivity than their heterogeneous counterparts [27], our aim was to carry out the catalytic reaction homogeneously. The solubility of the lipophilic catalyst **2** was investigated in ten solvents with low polarity, including a new, bio-based polar aprotic solvent, MeSesamol [33]. The catalyst’s precipitation – which is necessary for its recycling – was tested by adding a polar solvent, i.e., methanol, to its solution.

**Table 1:** Catalyst **2** solubility in solvents with low polarity.^a^

Solvent	Dissolved?	Precipitated by MeOH?

anisole	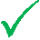	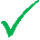
butyl acetate	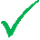	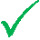
cyclohexane^b^	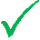	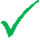
cyclopentyl methyl ether (CPME)	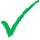	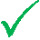
dichloromethane (DCM)	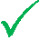	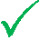
dimethyl carbonate (DMC)	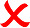	**–**
heptane	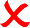	**–**
MeSesamol [33]	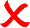	**–**
2-methyltetrahydrofuran (2-MeTHF)	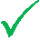	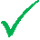
toluene	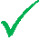	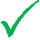

^a^To the lipophilic catalyst (2 mg) in a vial the appropriate solvent (200 µL) was added. Then, to check the precipitability of the catalyst, MeOH (800 µL) was added. ^b^Larger amounts of cyclohexane (1 mL) was needed to dissolve the catalyst.

The main requirement for the polar solvent is that it should not dissolve the catalyst while it should completely dissolve the product. Therefore, we investigated the solubility of the Michael adduct **14** in methanol, ethanol, propan-2-ol, Patosolv^®^ (a mixture of 85% of ethanol and 15% of propan-2-ol), and acetonitrile. The highest solubility of **14** was found in acetonitrile (63 mg mL^−1^) and methanol (17 mg mL^−1^). In both of these solvents, a low solubility of the lipophilic catalyst **2** was measured (<0.5 mg mL^−1^). Based on these results, acetonitrile was chosen as a precipitating solvent for the catalyst recycling.

To investigate the solvent effect, the stereoselective Michael addition reaction was carried out in those solvents that dissolved the catalyst and from which the catalyst was successfully precipitated by adding methanol. Furthermore, a reaction in which acetylacetone (**13**) did not only act as a reactant but also as a solvent was examined (Table 2).

**Table 2:** Solvent screening in the Michael addition of acetylacetone (**13**) to *trans*-β-nitrostyrene (**12**).^a^

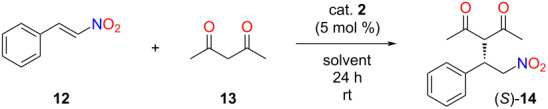			

Entry	Solvent	Yield^b^ [%]	ee^c^ [%]

1	CPME	85	91
2	toluene	88	93
3	DCM	91	93
4	acetylacetone	89	88
5	butyl acetate	22	94
6	2-MeTHF	94	87
7	anisole	84	89

^a^Reaction conditions: acetylacetone (**13**, 0.21 mmol) was added to the solution of *trans*-β-nitrostyrene (**12**, 0.08 mmol) and 5 mol % of catalyst **2** in 0.5 mL of solvent, then, the resulting mixture was stirred at room temperature for 24 hours. ^b^Isolated yields. ^c^Determined by chiral HPLC ((*S*)-enantiomer).

**Figure 2:**
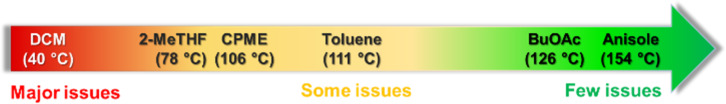
Classification of the tested non-polar solvents according to the GSK’s solvent sustainability guide [34].

The highest yields and enantiomeric excess values were reached in CPME, toluene, and dichloromethane (Table 2, entries 1–3). When acetylacetone was used as solvent, the enantiomeric excess was slightly lower because the catalyst was not completely dissolved in acetylacetone, which resulted in a heterogeneous reaction mixture (Table 2, entry 4). The importance of correct solvent selection is illustrated by the case of butyl acetate, in which only a low yield was observed (Table 2, entry 5). During solvent selection, both the catalytic performance and green chemistry aspects were addressed. For this purpose, we followed GSK’s solvent sustainability guide [34], which ranks solvents according to their properties, such as waste generation, environmental and health impacts, and boiling point (Figure 2).

**Figure 3:**
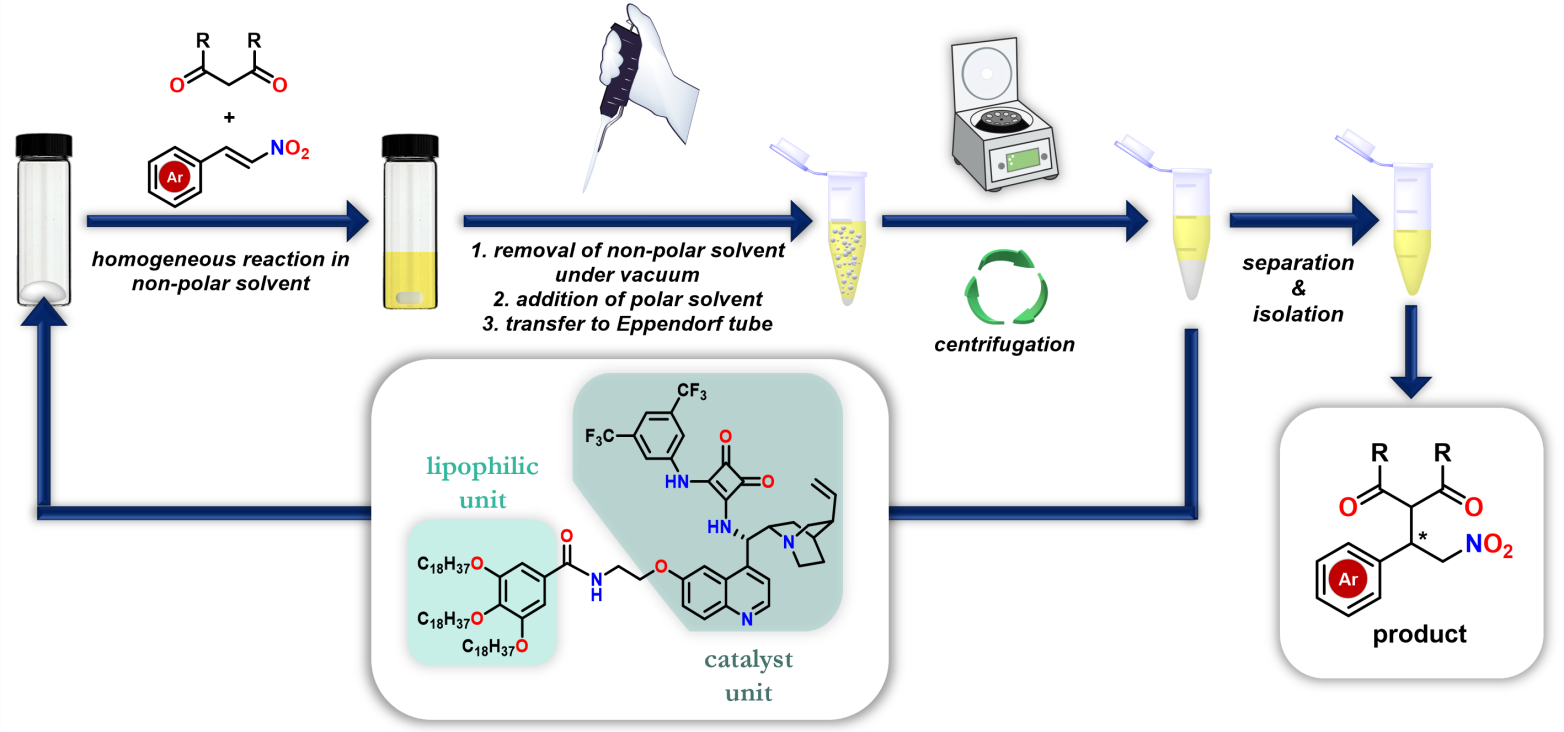
Recycling of the lipophilic organocatalyst in the stereoselective Michael addition by replacing the solvent.

Considering the three factors mentioned above (yield, enantiomeric excess, and green chemistry), toluene was chosen as a solvent for the recycling reactions. The schematic for the recycling by solvent replacement is shown in Figure 3.

After the stereoselective Michael addition was completed in non-polar toluene, toluene was evaporated in vacuo and then a polar solvent, acetonitrile was added, leading to the precipitation of the lipophilic catalyst but dissolution of the other reaction components. The reaction mixture was then transferred to Eppendorf vials and centrifuged (8 min, 13500 rpm). After phase separation, the product was isolated from the supernatant by preparative thin-layer chromatography, while the catalyst was reused in subsequent reaction cycles. The precipitated catalyst was further washed twice with acetonitrile, and dried in vacuo. The results are collected in Table 3.

**Table 3:** Recycling of lipophilic organocatalyst **2** in the Michael addition of acetylacetone (**13**) to *trans*-β-nitrostyrene (**12**).^a^

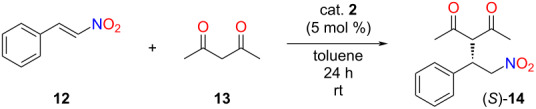			

Round	Yield^b^ [%]	ee^c^ [%]	Catalyst recycling efficiency [%]

1	84	91	91
2	89	90	99
3	94	91	92
4	93	91	99
5	96	92	97

^a^Reaction conditions: acetylacetone (**13**, 0.96 mmol) was added to the solution of *trans-*β-nitrostyrene (**12**, 0.38 mmol) and 5 mol % of catalyst **2** in 2 mL of toluene, then, the resulting mixture was stirred at room temperature for 24 hours. After the reaction was completed, the volatile components were evaporated, and acetonitrile was added for the recycling of the catalyst **2**. ^b^Isolated yields. ^c^Determined by chiral HPLC ((*S*)*-*enantiomer).

### Application and recycling of the lipophilic cinchona-squaramide organocatalyst in the synthesis of a baclofen precursor

**Scheme 3:**
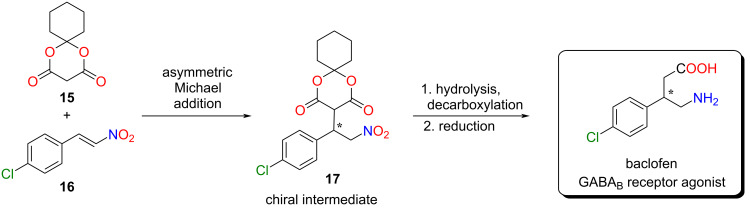
A new, stereoselective synthetic route for baclofen.

After the successful application of the lipophilic catalyst, its catalytic activity was also investigated in another industrially relevant stereoselective Michael addition. This type of reaction could be used in the synthesis of several drugs to form a carbon–carbon bond in a stereoselective manner [6,35]. Our goal was to synthesize the chiral precursor **17** of baclofen (Scheme 3).

To achieve this objective, we first planned to use Meldrum’s acid and 4-chloro-*trans*-β-nitrostyrene (**16**). Based on the literature [36], Meldrum’s acid has a low solubility in non-polar solvents, resulting in diminished enantioselectivity. Furthermore, in our case, the application of polar solvents would not be favorable due to the low solubility of the lipophilic organocatalyst in these solvents. Consequently, we applied the cyclohexyl derivative of Meldrum’s acid **15**, which exhibits enhanced solubility in non-polar solvents [36]. The Meldrum’s acid derivative **15** was synthesized from malonic acid and cyclohexanone using acetic anhydride and sulfuric acid as a catalyst (see Supporting Information File 1) [37].

In a similar manner as the former Michael addition, a solvent screening was carried out in the previously well-established four solvents: CPME, 2-MeTHF, anisole, and toluene (Table 4, entries 1–4).

**Table 4:** Solvent and catalyst amount screening in the Michael addition of the cyclohexyl derivative of Meldrum’s acid **15** to 4-chloro-*trans*-β-nitrostyrene (**16**).^a^

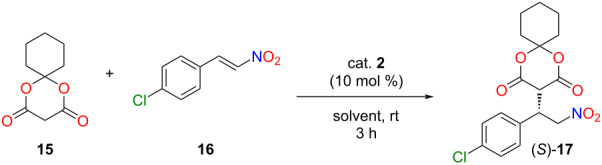				

Entry	Solvent	Catalyst amount [mol%]	Yield^b^ [%]	ee^c^ [%]

1	CPME	10	93	82
2	2-MeTHF	10	93	29
3	anisole	10	>99	93
4	toluene	10	>99	92
5	toluene	5	97	93
6^d^	toluene	1	94	96

^a^Reaction conditions: Meldrum’s acid derivative **15** (0.064 mmol) was added to the solution of 4-chloro-*trans*-β-nitrostyrene (**16**, 0.096 mmol) and 1, 5 or 10 mol % of catalyst **2** in 470 µL of solvent, then, the resulting mixture was stirred at room temperature for 3 hours. ^b^Isolated yields. ^c^Determined by chiral HPLC ((*S*)-enantiomer). ^d^Reaction time was 5 hours to achieve full conversion.

Based on the solvent screening, high yields and good enantioselectivity can be achieved in anisole and toluene, while using 2-MeTHF drastically decreased the enantioselectivity. From the perspective of green chemistry, anisole would be more favorable, but toluene is also a distinctly better alternative to the other solvents (e.g., DCM) commonly used in Michael additions. Another critical aspect that needs to be considered is how difficult the removal of the solvent is. In this respect, the boiling point of toluene (111 °C) is preferable to that of anisole, which has a boiling point of 154 °C. The results of catalyst recycling in anisole and toluene are shown in Table 5.

**Table 5:** Recycling of lipophilic organocatalyst **2** in the Michael addition of the cyclohexyl derivative of Meldrum’s acid **15** to 4-chloro-*trans*-β-nitrostyrene (**16**).^a^

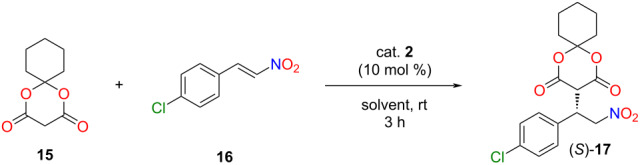			

Round	Solvent	Yield^b^ [%]	ee^c^ [%]

1	anisole	95	92
2		>99	95
3		>99	94
4		>99	93
5		>99	93
1	toluene	94	93
2		>99	94
3		>99	94
4		>99	94
5		97	92

^a^Reaction conditions: Meldrum’s acid derivative **15** (0.19 mmol) was added to the solution of 4-chloro-*trans*-β-nitrostyrene (**16**, 0.29 mmol) and 10 mol % of catalyst **2** in 1.4 mL of solvent, then, the resulting mixture was stirred at room temperature for 3 hours. After the reaction was completed, the volatile components were evaporated, and acetonitrile was added for the recycling of the catalyst **2**. ^b^Isolated yields. ^c^Determined by chiral HPLC ((*S*)-enantiomer).

The lipophilic organocatalyst maintained its activity in both solvents over the five reaction cycles while the catalyst loss was marginal (<10%). It is important to note that in an industrial-scale process, the catalyst loss could be further diminished, and the centrifugation could be replaced by a simple filtration. Furthermore, for large-scale applications, the effect of catalyst amount was also investigated (Table 4, entries 4–6). Yields and enantiomeric excess values did not change significantly when the catalyst amount was reduced from 10 mol % to 1 mol %, however, a longer reaction time (5 hours) was required for the 1 mol % catalyst loading.

### Gram-scale synthesis of baclofen

Finally, we planned to demonstrate the recyclability of our lipophilic organocatalyst **2** in the gram-scale synthesis of baclofen precursor (*S*)-**17**. The catalyst loading was set to 1 mol % to reduce the needed catalyst amount and the reaction time was increased to 5 hours to achieve full conversion. After the organocatalytic reaction in toluene, the volatile components were evaporated, and acetonitrile was added to precipitate the catalyst. In contrast to the small-scale recycling, in this case, we used filtration instead of centrifugation to recover the catalyst without significant loss (<5%). This demonstrates that we developed an organocatalytic reaction that can be easily scaled-up and the novel lipophilic catalyst can be recovered not only by centrifugation but also by filtration.

**Scheme 4:**
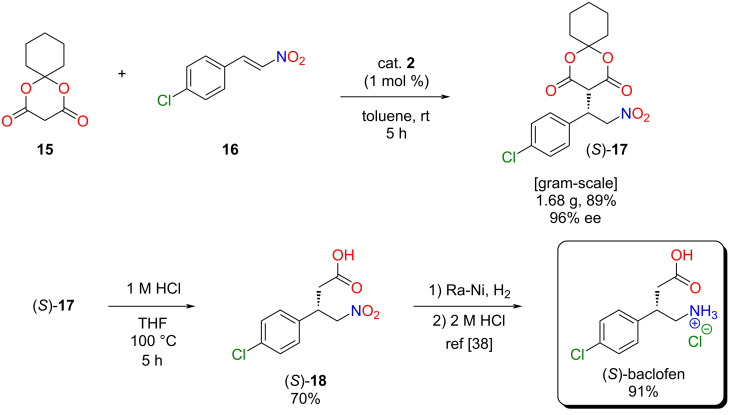
Gram-scale synthesis of (*S*)-baclofen hydrochloride.

From the baclofen precursor (*S*)-**17**, baclofen can be synthesized in two steps. The nitro acid (*S*)-**18** was obtained using HCl in THF in good yield (70%), which could be reduced to (*S*)-baclofen hydrochloride using Raney nickel as catalyst (Scheme 4) [38].

## Conclusion

In conclusion, we have prepared a new lipophilic cinchona squaramide organocatalyst **2** modified with octadecyl side chains. Thanks to the lipophilic unit, the catalyst can be easily precipitated by exchanging the non-polar solvent with a more polar one, and then its separation can be achieved by centrifugation. The lipophilic catalyst **2** demonstrated its excellent catalytic activity in two stereoselective Michael addition reactions. Homogeneous catalysis was carried out in non-polar solvents (i.e., toluene), which allows the high performance of the lipophilic organocatalyst in terms of yield and stereoselectivity. To facilitate the pharmaceutical use of the lipophilic organocatalyst, we investigated a new, industry-relevant synthesis route of baclofen in the gram-scale. The chiral precursor **17** of baclofen was obtained with quantitative yield and excellent enantiomeric excess (up to 96%). The catalyst was applied in five consecutive runs without a decrease in catalytic activity, moreover, the catalyst loss was also negligible (<10%). Overall, it can be concluded that the incorporation of the lipophilic unit does not affect the catalytic activity and selectivity but enables the facile recycling of the catalyst.

## Experimental

### General information

The starting materials and reagents were purchased from commercially available sources (Merck, TCI Europe, and VWR). Infrared (IR) spectra were recorded on a Bruker Alpha-T Fourier-transform IR (FTIR) spectrometer. Optical rotations were measured on a Perkin Elmer 241 polarimeter calibrated by measuring the optical rotations of both enantiomers of menthol. The reactions under pressure were carried out in a 150 mL pressure flask (Synthware Glass). Thin-layer chromatography (TLC) was performed using silica gel 60 F_254_ (Merck) plates. The spots of the materials on TLC plates were visualized by UV light at 254 nm. The reactions were monitored by TLC and high-performance liquid chromatography–mass spectrometry (HPLC–MS). The solvent ratios of the eluents are given in volume units (mL mL^−1^). Nuclear magnetic resonance (NMR) spectra were recorded on a Bruker DRX-500 Avance spectrometer (at 500 and 126 MHz for the ^1^H and ^13^C spectra, respectively) or on a Bruker 300 Avance spectrometer (at 300 and 75.5 MHz for the ^1^H and ^13^C spectra, respectively) or on a Bruker Avance III HD (at 600 MHz for ^1^H and at 150 MHz for ^13^C spectra) at specified temperatures. High-resolution MS was measured on a Bruker MicroTOF II instrument using positive electrospray ionization. HPLC–MS was performed on an HPLC system using a Shimadzu LCMS-2020 (Shimadzu Corp., Japan) device equipped with a Reprospher (Altmann Analytik Corp., Germany) 100 Å C18 (5 µm; 100 × 3 mm) column and a positive/negative double ion source with a quadrupole MS analyzer in the range of 50–1000 *m*/*z*. Further details are available in Supporting Information File 1. The enantiomeric ratios of the samples were determined by chiral high-performance liquid chromatography (HPLC) measurements. The exact conditions of chiral HPLC are indicated in the experimental section of the corresponding compound. MarvinSketch was used for log*P* prediction, MarvinSketch 20.11, ChemAxon (https://www.chemaxon.com).

### Demethylated cinchona squaramide **6**

The demethylated cinchona squaramide was prepared according to the literature procedure [39]. To the best of our knowledge, the NMR assignment has not been reported yet. ^1^H NMR (methanol-*d*_4_, 600 MHz, 295 K) δ 8.68 (d, ^3^*J*_H,H_ = 4.7 Hz, 1H), 8.01 (bs, 2H), 7.94 (d, ^3^*J*_H,H_ = 9.1 Hz, 1H), 7.72 (bs, 1H), 7.55 (m, 2H), 7.40 (dd, ^3^*J*_H,H_ = 9.1 Hz, ^4^*J*_H,H_ = 2.5 Hz, 1H), 6.15 (bs, 1H), 5.91 (m, 1H), 5.07 (bd, ^2^*J*_H,H_ = 17.2 Hz, 1H), 5.02 (bd, ^2^*J*_H,H_ = 10.4 Hz, 1H), 3.49 (m, 1H), 3.48 (m, 1H), 3.33 (m, 1H), 2.86 (m, 1H), 2.82 (m, 1H), 2.41 (bs, 1H), 1.69 (m, 1H), 1.68 (m, 2H), 1.54 (m, 1H), 0.85 (m, 1H); ^13^C NMR (methanol-*d*_4_, 150 MHz, 295 K) δ 185.7, 182.1, 170.4, 164.8, 158.3, 147.6, 145.3, 144.6, 142.5, 142.3, 133.9 (q, ^2^*J*_C,F_ = 33.4 Hz), 131.7, 129.7, 124.5 (q, ^1^*J*_C,F_ = 272.0 Hz), 123.9, 120.0, 119.3 (q, ^3^*J*_C,F_ = 3.0 Hz), 116.7 (m), 115.2, 105.4, 61.9, 56.9, 54.7, 41.8, 40.7, 28.8, 28.3, 26.9; for the full ^1^H and ^13^C assignment of demethylated cinchona squaramide structure, see Supporting Information File 1.

### Cinchona squaramide organocatalyst with linker, **7**


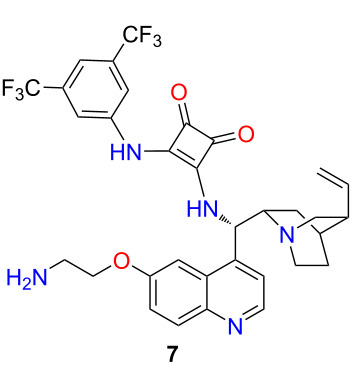


The demethylated cinchona squaramide **6** (732 mg, 1.19 mmol) was dissolved in DMF (15 mL), and caesium carbonate (1.75 g, 5.37 mmol, 4.5 equiv) was added to the solution at 0 °C. The reaction mixture was stirred for 20 min at this temperature. Then, the DMF (10 mL) solution of tosylated *N*-Boc-protected ethanolamine (955 mg, 3.03 mmol, 2.5 equiv, Scheme 2) was added dropwise. The reaction mixture was stirred at 50 °C for 8 hours. The volatile components were removed under reduced pressure. To the resulting orange oil, water (60 mL) was added, and the product was extracted with dichloromethane (3 × 60 mL). The combined organic phase was washed with water (2 × 60 mL), dried over MgSO_4,_ and concentrated in vacuo. The intermediate (1.24 g) was used in the next reaction without purification. The crude, *N*-Boc-protected intermediate was dissolved in dichloromethane (25 mL) and the solution cooled to 0 °C with an ice bath. Next, trifluoroacetic acid (5.74 mL, 75 mmol, 63 equiv) was added dropwise. The reaction mixture was stirred at room temperature for 1 h. Then, it was cooled to 0 °C, and a 40% NaOH(aq) solution was added to set the pH to 13. To this mixture, water (60 mL) was added, and it was extracted with DCM/MeOH 20:1 (60 mL). After the separation of the phases, the aqueous phase was extracted again with DCM/MeOH 20:1 (3 × 40 mL). The combined organic phase was dried over MgSO_4_ and concentrated in vacuo. The obtained residue (609 mg, 78% for 2 steps) was a dark orange solid, and we used this product in the next step without further purifications. TLC (SiO_2_; DCM/MeOH/25% NH_4_OH(aq) 10:1:0.01, *R*_f_ 0.22); mp 158–160 °C; 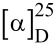 −51.8 (*c* 1.00, DMSO); IR (cm^−1^) ν_max_: 2957, 2923, 2853, 1664, 1620, 1609, 1560, 1508, 1437, 1378, 1331, 1277, 1201, 1175, 1126, 1021, 930, 884, 832, 799, 719, 700, 679, 620, 550, 521, 414; ^1^H NMR (500 MHz, MeOH-*d*_4_, 298 K) δ 8.77 (d, *J* = 4.7 Hz, 1H), 8.03–7.96 (m, 3H), 7.94 (bs, 1H), 7.69 (d, *J* = 4.7 Hz, 1H), 7.51 (dd, *J* = 2.57 Hz, 9.27 Hz, overlapped, 1H) 7.49 (bs, overlapped, 1H), 6.28 (d, *J* = 8.9 Hz, 1H), 5.95 (ddd, *J* = 17.5, 10.3, 7.7 Hz, 1H), 5.08 (bd, *J* = 17.5 Hz, 1H), 5.03 (bd, *J* = 10.3 Hz, 1H), 4.37 (m, 2H), 3.69–3.59 (m, 1H), 3.56–3.47 (m, 1H), 3.30–3.27 (m, overlapped, 1H), 2.99 (s, 1H), 2.93–2.86 (m, 1H), 2.86 (s, 1H), 2.84–2.76 (m, 1H), 2.43–2.37 (m, 1H), 1.70–1.61 (m, overlapped, 4H), 1.27 (m, 1H), 0.73–0.66 (m, 1H) ppm; ^13^C NMR (126 MHz, MeOH-*d*_4_, 298 K) δ 186.4, 182.8, 170.9, 165.9, 159.7, 149.3, 146.1, 146.0, 143.5, 143.0, 134.2 (q, *J* = 33.1 Hz), 132.4, 129.9, 125.1 (q, *J* = 272.1 Hz), 124.9, 120.3, 117.0, 115.8, 103.7, 69.6, 61.4, 57.5, 55.4, 42.2, 41.5, 41.2, 37.4, 32.1, 29.4, 29.0, 28.0 ppm.; HRESI(+)-MS (*m*/*z*): [M + H^+^] calcd for C_33_H_32_F_6_N_5_O_3_, 660.2409; found, 660.2424.

### Methyl 3,4,5-tris(octadecyloxy)benzoate (**9**)


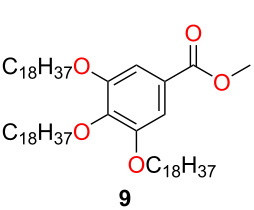


Methyl gallate (**8**, 15 g, 0.082 mol) was dissolved in DMF (150 mL), and 1-bromooctadecane (87.3 g, 0.262 mol) and potassium carbonate (67.5 g, 0.489 mol) were added. To the resulting reaction mixture, further DMF (150 mL) was added. After stirring for 16 hours at 80 °C, the reaction mixture was cooled, and diluted with toluene, water, and chloroform to precipitate the product. The crude product was filtered and recrystallized using chloroform/methanol mixed solvents. For a typical recrystallization of 1 gram crude product, 30 mL chloroform and 90 mL methanol were applied. The recrystallized solid was filtered to obtain product **9** as a white solid (49.2 g, 64%). The products had the same spectroscopic data than those reported in the literature [32].

### Lipophilic organocatalyst **2**


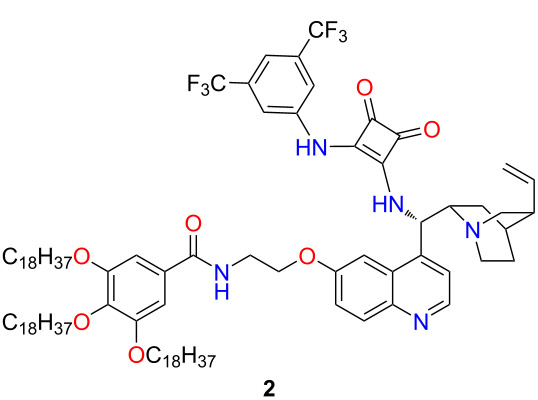


The cinchona squaramide with the aminoethylene linker **7** (534 mg, 0.81 mmol, 1.05 equiv) and triethylamine (1.09 mL, 8.26 mmol, 10.2 equiv) were dissolved in dichloromethane (8.8 mL). Then, a solution of 3,4,5-tris(octadecyloxy)benzoyl chloride (**11**, 729.3 mg, 0.77 mmol) in dichloromethane (22 mL) was added. To the resulting yellow solution further dichloromethane (12.6 mL) was added, and the mixture was stirred for 24 hours. Next, the reaction mixture was washed with water (2 × 30 mL), and the organic phase was dried over MgSO_4_, and concentrated in vacuo. The crude product was purified by column chromatography (SiO_2_, DCM/MeOH/NH_3_(aq) 20:1:0.01 → DCM/MeOH/NH_3_ 10:1:0.01). The product was dissolved in a small amount of dichloromethane (1 mL) and added dropwise to acetonitrile (100 mL) to precipitate the product as an off-white solid (698 mg, 58%). TLC (SiO_2_, DCM/MeOH 20:1, *R*_f_ 0.35); mp 68–69 °C; 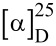 +14.1 (*c* 1.00, CHCl_3_); IR (cm^−1^) ν_max_: 3267, 2916, 2850, 1792, 1689, 1622, 1604, 1587, 1551, 1466, 1437, 1378, 1335, 1277, 1226, 1181, 1133, 1117, 1047, 1000, 930, 879, 851, 821, 764, 721, 701, 680, 619, 526; ^1^H NMR (500 MHz, CDCl_3_, 318 K) δ 8.73 (d, *J* = 3.6 Hz, 1H), 8.03 (m, 1H), 7.90–7.62 (m, 2H), 7.57–7.42 (m, overlapped, 1H), 7.40–7.33 (m, overlapped, 3H), 7.28 (m, 1H), 7.08 (br s, 2H), 6.30 (br s, 1H), 5.70 (m, 1H), 5.21–4.90 (m, 2H), 4.45 (m, 2H), 4.06–3.88 (m, overlapped, 10H), 3.76–3.69 (m, 2H), 3.61–3.19 (m, 2H), 3.09–2.65 (m, 2H), 1.93–1.61 (m, overlapped, 7H), 1.52–1.11 (m, overlapped, 94H), 0.88 (t, *J* = 7.0 Hz, 9H,) ppm; ^13^C NMR (75 MHz, CDCl_3_, 318 K) δ 185.4, 180.3, 175.4, 168.3, 165.1, 157.8, 153.3, 153.0, 147.9, 145.1, 141.6, 141.4, 140.3, 132.7 (q, ^2^*J*_C,F_ = 34.2 Hz), 132.3, 130.3, 129.0, 127.6, 122,9 (q, ^1^*J*_C,F_ = 272.0 Hz), 118.4, 116.5, 116.1, 108.0, 105.9, 101.3, 73.7, 73.6, 69.5, 69.2, 66.7, 60.4, 53.5, 41.2, 39.1, 32.1, 30.5, 29.9, 29.8, 29.6, 29.5, 26.3, 26,2 22.8, 14.2 ppm; ^19^F NMR −63.1 ppm; HRESI(+)-MS (*m*/*z*): [M + H^+^] calcd for C_94_H_144_F_6_N_5_O_7_, 1569.0970; found, 1569.957.

### General procedure for the solvent screening of stereoselective Michael addition of acetylacetone to *trans*-β-nitrostyrene


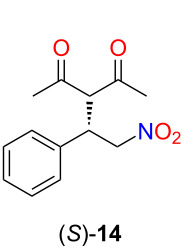


First, *trans*-β-nitrostyrene (**12**, 11.9 mg, 0.08 mmol) and the lipophilic organocatalyst **2** (6.3 mg, 0.004 mmol, 5 mol %) were dissolved in the appropriate solvent (500 µL). Then, acetylacetone (**13**, 21.1 μL, 0.205 mmol) was added. The reaction mixture was stirred for 24 hours at room temperature. After the reaction was completed, the solvent was evaporated, and the crude product was purified by preparative thin-layer chromatography (SiO_2_, hexane/ethyl acetate 2:1, *R*_f_ 0.36) to obtain the product (*S*)-**14** as pale-yellow crystals. The products had the same spectroscopic data than those of reported (the absolute configuration was determined by the optical rotation of the products) [26]. HPLC: Phenomenex Lux Cellulose-3 column (3 mm, 250 × 4.6 mm), eluent CH_3_CN/H_2_O (0.1% formic acid) 40:60, isocratic mode; 0.6 mL min^−1^; UV detector 222 nm, 30 °C. The retention time for the (*S*)-enantiomer was 12.3 min, for the (*R*)-enantiomer 14.7 min.

### General procedure for recycling of the lipophilic organocatalyst in the stereoselective Michael addition of acetylacetone to *trans*-β-nitrostyrene

*trans*-β-Nitrostyrene (**12**, 57 mg, 0.382 mmol) and the lipophilic organocatalyst **2** (30 mg, 0.0191 mmol, 5 mol %) were dissolved in toluene (2.0 mL), and acetylacetone (**13**, 99 μL, 0.962 mmol) was added. The reaction mixture was stirred for 24 hours at room temperature. Then, the volatile components were evaporated, and acetonitrile (2 mL) was added. The dissolution of the product and the suspension of the catalyst were aided by using an ultrasonic bath. The reaction mixture was transferred to Eppendorf tubes, and the insoluble components were separated by centrifugation (8 min, 13500 rpm). After the separation, the precipitated catalyst was similarly washed twice with acetonitrile (2 × 2 mL). The combined acetonitrile phase was evaporated, and the crude product was purified by using preparative thin-layer chromatography (SiO_2_, hexane/ethyl acetate 2:1, *R*_f_ 0.36) to obtain the product (*S*)-**14**. The catalyst was dried in vacuo and reused in the following reaction cycle.

### General procedure for the solvent screening of stereoselective Michael addition of the cyclohexyl derivative of Meldrum’s acid to 4-chloro-*trans*-β-nitrostyrene


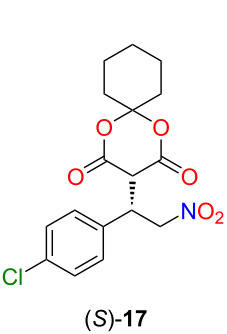


Cyclohexyl derivative of Meldrum’s acid **15** (11.7 mg, 0.064 mmol), 4-chloro-*trans*-β-nitrostyrene (**16**, 17.5 mg, 0.096 mmol), and the lipophilic organocatalyst **2** (1, 5, or 10 mol %) were dissolved in the appropriate solvent (470 µL), and stirred for 3 hours at room temperature. Then, the solvent was evaporated, and the crude product was purified by preparative thin-layer chromatography (SiO_2_, hexane/ethyl acetate/AcOH 2:1:0.01, *R*_f_ 0.34) to obtain the product as a pale-yellow foam. To the best of our knowledge, the synthesis of (*S*)-**17** has not been reported so far. TLC (SiO_2_, hexane/acetone 1:3, *R*_f_ 0.85); mp 61–69 °C; 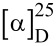 −5.7 (*c* 1.00, CHCl_3_); IR (cm^−1^) ν_max_ 2941, 2860, 1780, 1744, 1709, 1637, 1553, 1493, 1448, 1433, 1416, 1367, 1339, 1299, 1272, 1223, 1184, 1133, 1091, 1068, 1036, 1014, 998, 976, 956, 912, 49, 825, 783, 743, 718, 679, 654, 530, 425; ^1^H NMR (500 MHz, CDCl_3_, 298 K) δ 7.33–7.28 (m, 4H), 5.35 (dd, *J* = 13.9, 8.7 Hz, 1H), 5.00 (dd, *J* = 13.9, 6.7 Hz, 1H), 4.63 (ddd, *J* = 8.8, 6.7, 3.1 Hz, 1H), 4.02 (d, *J* = 3.2 Hz, 1H), 1.91 (t, *J* = 6.2 Hz, 2H), 1.67 (m, overlapped, 2H), 1.48–1.42 (m, 2H), 1.30–1.26 (m, overlapped, 4H) ppm; ^13^C NMR (75 MHz, CDCl_3_, 298 K) δ 164.4, 163.9, 135.1, 133.7, 130.7, 129.5, 106.9, 76.1, 49.0, 41.4, 36.8, 36.7, 29.8, 21.8 ppm; HRESI(+)-MS (*m*/*z*): [M + Na^+^] calcd for C_17_H_18_ClNO_6_Na, 390.0720; found, 390.0682; HPLC: Phenomenex Lux Cellulose-3 column (3 mm, 250 × 4.6 mm), eluent CH_3_CN/H_2_O (0.2% formic acid) 40:60, isocratic mode; 1 mL min^−1^; UV detector 265 nm, 35 °C. The retention time for the (*S*)-enantiomer was 27.2 min, for the (*R*)-enantiomer 29.1 min.

### General procedure for recycling of the lipophilic organocatalyst in the stereoselective Michael addition of the cyclohexyl derivative of Meldrum’s acid to 4-chloro-*trans*-β-nitrostyrene

Cyclohexyl derivative of Meldrum’s acid **15** (35.2 mg, 0.19 mmol), 4-chloro-*trans*-β-nitrostyrene (**16**, 52.7 mg, 0.29 mmol), and the lipophilic organocatalyst **2** (30 mg, 0.0019 mmol, 10 mol %) were dissolved in toluene or anisole (1.4 mL), and stirred for 3 hours at room temperature. After the reaction was completed, the solvent was evaporated, and acetonitrile (1.5 mL) was added. The dissolution of the product and the suspension of the catalyst were aided by using an ultrasonic bath. The reaction mixture was transferred to Eppendorf tubes, and the insoluble components were separated by centrifugation (8 min, 13500 rpm). After the separation, the precipitated catalyst was similarly washed twice with acetonitrile (2 × 1.5 mL). The combined acetonitrile phase was evaporated, and the crude product was purified by using preparative thin-layer chromatography (SiO_2_, hexane/ethyl acetate/AcOH 2:1:0.01, *R*_f_ 0.34) to obtain the product (*S*)-**17**. The catalyst was dried in vacuo and reused in the following reaction cycle.

### Gram-scale synthesis of baclofen precursor **17**

Cyclohexyl derivative of Meldrum’s acid **15** (938.4 mg, 5.1 mmol), 4-chloro-*trans*-β-nitrostyrene (**16**, 1404.2 mg, 7.6 mmol), and the lipophilic organocatalyst **2** (80 mg, 0.05 mmol, 1 mol %) were dissolved in toluene or anisole (37.6 mL), and stirred for 5 hours at room temperature. After the reaction was completed, the solvent was evaporated, and acetonitrile (30 mL) was added. The dissolution of the product and the suspension of the catalyst were aided by using an ultrasonic bath. The resulting solid was filtrated, and washed with acetonitrile (3 × 10 mL). The combined acetonitrile phase was evaporated, and the crude product was purified by using column chromatography (SiO_2_, heptane/ethyl acetate/AcOH 4:1:0.01) to obtain the product ((*S*)-**17**, 1.675 g, 89%).

### Synthesis of nitro acid (*S*)-**18**


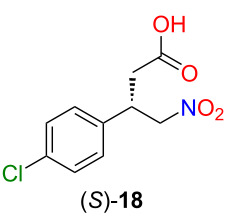


In a 150 mL pressure flask (Synthware), the baclofen precursor (*S*)-**17** (0.5 g, 1.36 mmol) was dissolved in THF (71 mL), and 1 M HCl (aq. solution, 36 mL) was added. The reaction mixture was heated in a 100 °C oil bath for 5 hours. Then, the volatile components were evaporated, and the aqueous phase was extracted with EtOAc (3 × 10 mL). The combined organic phase was dried over MgSO_4_ and concentrated in vacuo. The crude product was purified by column chromatography (SiO_2_, heptane/ethyl acetate/AcOH 4:1:0.01 → heptane/ethyl acetate/AcOH 1:1:0.01) to gain the product (*S*)-**18** as a white solid (232 mg, 70%). TLC (SiO_2_, heptane/ethyl acetate/AcOH 1:1:0.01, *R*_f_ 0.52, visualized by bromocresol green); mp 82–84 °C (lit. [40] 78–80 °C); 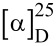 −10.5 (*c* 2.00, MeOH), (lit. [38]: 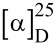 −10.1 (*c* 2.00, MeOH)). The products had the same spectroscopic data than those reported in the literature [41].

## Supporting Information

File 1 Experimental part, NMR, IR, and chiral HPLC spectra.
