# Supplementary material for: A novel recyclable organocatalyst for the gram-scale enantioselective synthesis of (S)-baclofen
Source: Beilstein J Org Chem. 2023 Nov 24;19:1811–24. doi: 10.3762/bjoc.19.133 (PMC10682516; doi:10.3762/bjoc.19.133)
Supplement: File 1 — Experimental part, NMR, IR, and chiral HPLC spectra. [file Beilstein_J_Org_Chem-19-1811-s001.pdf]

BEILSTEIN JOURNAL OF ORGANIC CHEMISTRY

Supporting Information

for

A novel recyclable organocatalyst for the gram-scale enantioselective synthesis of (*S*)-baclofen

Gyula Dargó, Dóra Erdélyi, Balázs Molnár, Péter Kisszékelyi, Zsófia Garádi and József Kupai

Beilstein J. Org. Chem. **2023,** *19,* 1811–1824. doi:10.3762/bjoc.19.133

Experimental part, NMR, IR, and chiral HPLC spectra

License and Terms: This is a supporting information file under the terms of the Creative Commons Attribution License (https://creativecommons.org/licenses/by/4.0). Please note that the reuse, redistribution and reproduction in particular requires that the author(s) and source are credited and that individual graphics may be subject to special legal provisions.
The license is subject to the *Beilstein Journal of Organic Chemistry* terms and conditions: (https://www.beilstein-journals.org/bjoc/terms)

Table of contents

Experimental procedure for the synthesis of Meldrum's acid derivative **15** S1
Catalyst **2** solubility in solvents with low polarity S1
The solubility of the Michael adduct (*S*)-**14** in polar solvents S2
Chiral HPLC profiles of Michael adduct **14** S2
Chiral HPLC profiles of baclofen intermediate **17** S3
NMR spectra of demethylated cinchona squaramide **6** S4
NMR spectra of cinchona squaramide organocatalyst with linker **7** S10
FT-IR spectrum of cinchona squaramide with linker **7** S11
NMR spectra of lipophilic organocatalyst **2** S12
FT-IR spectrum of lipophilic organocatalyst **2** S16
NMR spectra of chiral baclofen intermediate **17** S17
FT-IR spectrum of chiral baclofen intermediate **17** S18

Experimental procedure for the synthesis of Meldrum's acid derivative 15

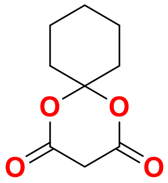

15

The cyclohexyl derivative of Meldrum's acid **15** was obtained by following a literature procedure[1] with minor modifications. Malonic acid (10.0 g, 96 mmol) was dissolved in acetic anhydride (9.6 mL) and three drops of 98% sulfuric acid were added. The mixture was heated to 60 °C and stirred at this temperature for 15 minutes. The reaction mixture was then cooled to room temperature, and cyclohexanone (9.9 mL, 96 mmol) was added dropwise over 45 min. The reaction mixture was stirred for further 20 hours. Next, the volatile components were evaporated, and the brown, oily evaporation residue was dissolved in diethyl ether (80 mL) and washed with water (3 × 15 mL). The organic phase was dried with $MgSO_4$ and concentrated in vacuo until the total volume of the solution was 5 mL. After the addition of hexane, the mixture was placed in the freezer, which resulted in the precipitation of the pale yellow product. The precipitate was filtered and washed with hexane to obtain the crude product, which was further purified by column chromatography (SiO_2, Hex/EtOAc 4:1 → Hex/EtOAc 2:1). Yield: 4.08 g (23%)

Catalyst 2 solubility in solvents with low polarity

To the lipophilic catalyst (2 mg) in a vial, the appropriate solvent (200 µL) was added. Then, to check the precipitability of the catalyst, MeOH (800 µL) was added, where the opalescence of the solution indicated that the catalyst could be precipitated from the solvent. In the case of cyclohexane, a larger amount of solvent (1 mL) was added to gain a slightly opalescent solution. DMC, heptane, and MeSesamol did not dissolve the lipophilic catalyst even with a larger amount (500 µL) of solvent. Furthermore, it is important to note that cyclohexane and heptane are not miscible with MeOH, thus, for the precipitation, a less polar solvent (e.g., propan-2-ol) might be used.

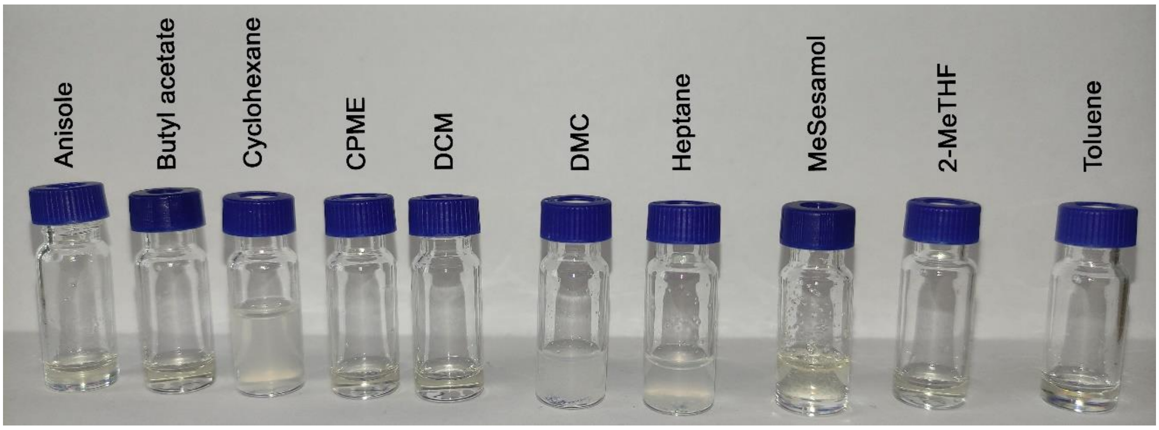

Figure S1: Solubility tests of the lipophilic catalyst **2** in different solvents.

[1] Jiang, H.; Zhang, J. M.; Du, W. Q.; Zhu, S. Z. *Chin. J. Chem.* **2007**, *25*, 86–89. DOI: 10.1002/CJOC.200790023.

The solubility of the Michael adduct (*S*)-14 in polar solvents

The appropriate polar solvent (EtOH, Patosolv®, MeOH, propan-2-ol, acetonitrile) was added to Michael adduct (*S*)-**14** (10 mg) until it was completely dissolved.

Solvent	Solubility
acetonitrile	62.5 mg mL^{-1}
MeOH	16.7 mg mL^{-1}
EtOH	7.0 mg mL^{-1}
Patosolv® (85% EtOH, 15% IPA)	6.7 mg mL^{-1}
propan-2-ol	3.0 mg mL^{-1}

Chiral HPLC profiles of Michael adduct 14

Chiral HPLC: Phenomenex Lux Cellulose-3 column (3 mm, 250 × 4.6 mm), eluent CH_3CN/H_2O (0.1% formic acid) 40:60, isocratic mode; 0.6 mL min^{-1}; UV detector 222 nm, 30 °C. The retention time for the (*S*)-enantiomer was 12.3 min, for the (*R*)-enantiomer, 14.7 min.

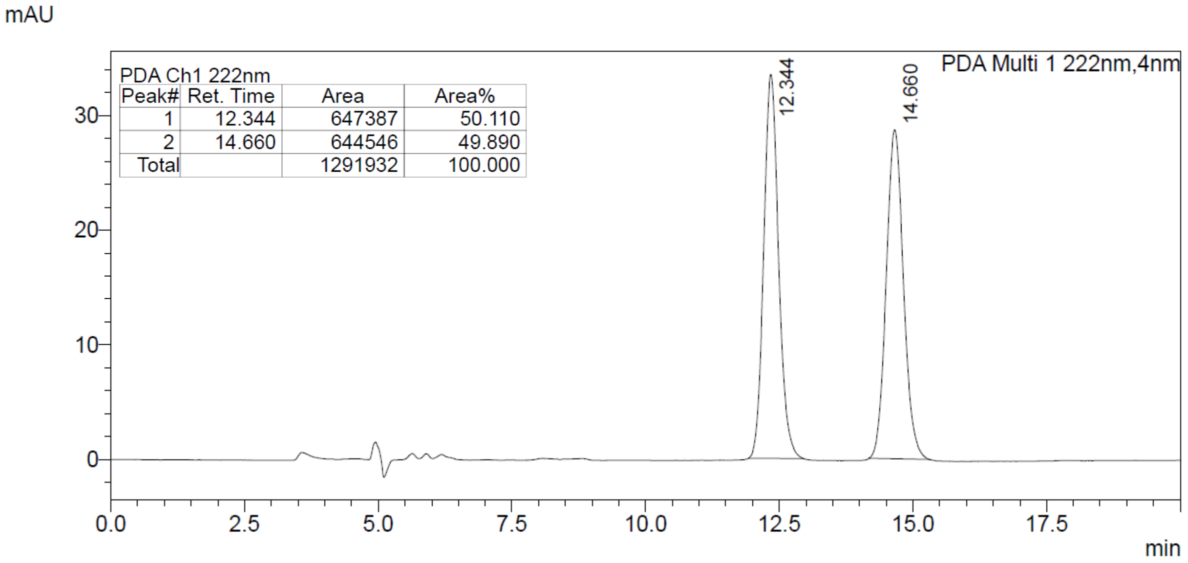

Figure S2 Chiral HPLC chromatogram of racemic Michael adduct **14**

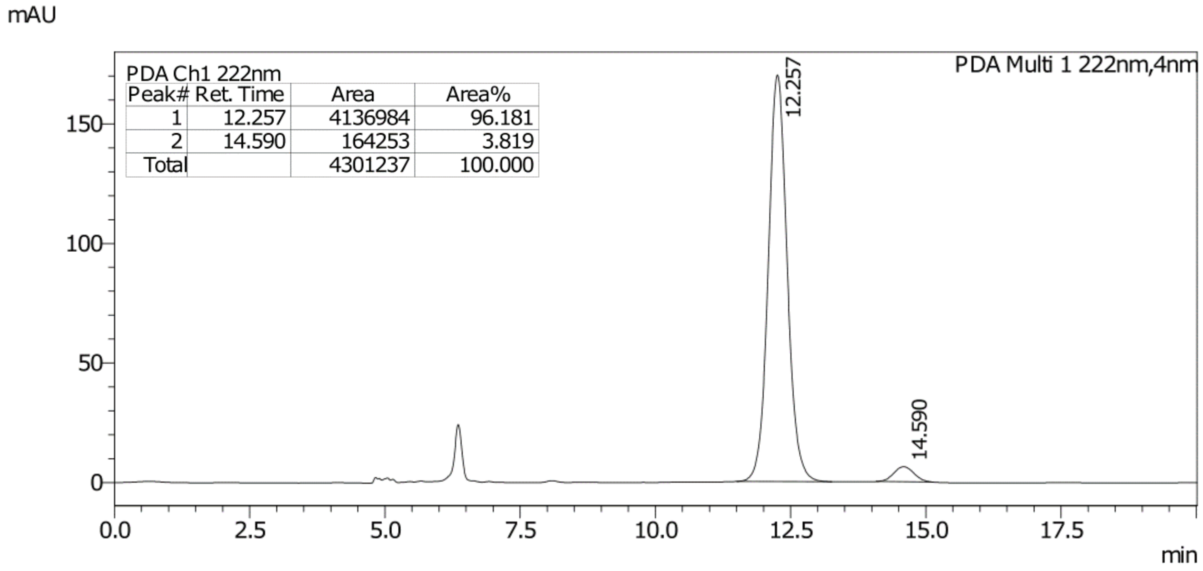

Figure S3 Chiral HPLC chromatogram of Michael adduct **14** (Table 3, round 5)

Chiral HPLC profiles of baclofen intermediate 17

Chiral HPLC: Phenomenex Lux Cellulose-3 column (3 mm, 250 × 4.6 mm), eluent CH_3CN/H_2O (0.2% formic acid) 40:60, isocratic mode; 1 mL min^{-1}; UV detector 265 nm, 35 °C. The retention time for the (*S*)-enantiomer was 27.2 min, for the (*R*)-enantiomer, 29.1 min.

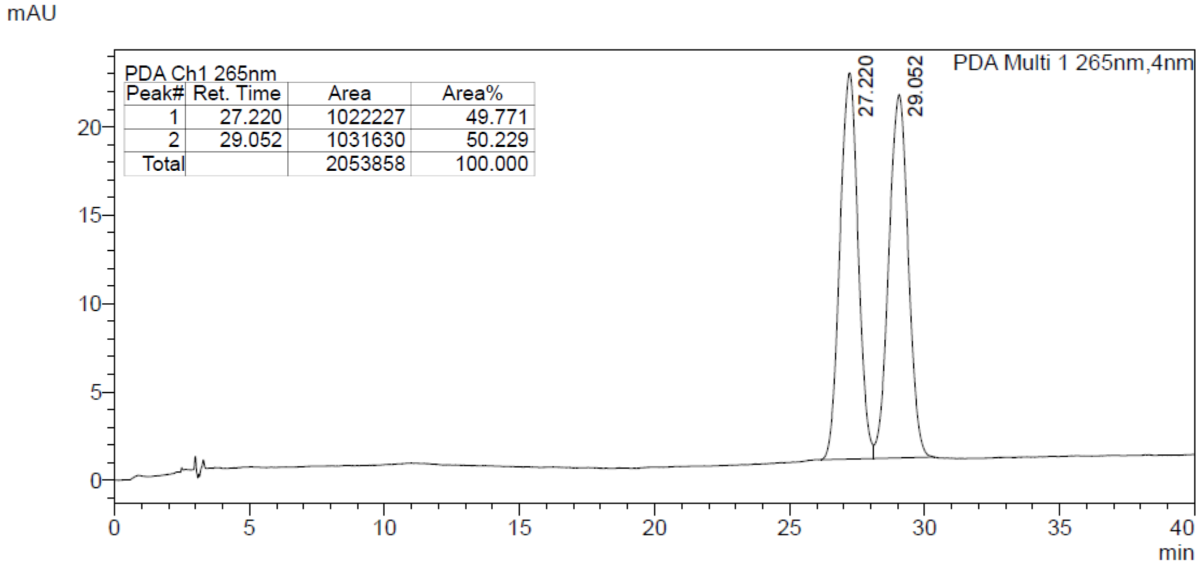

Figure S4 Chiral HPLC chromatogram of racemic baclofen intermediate **17**

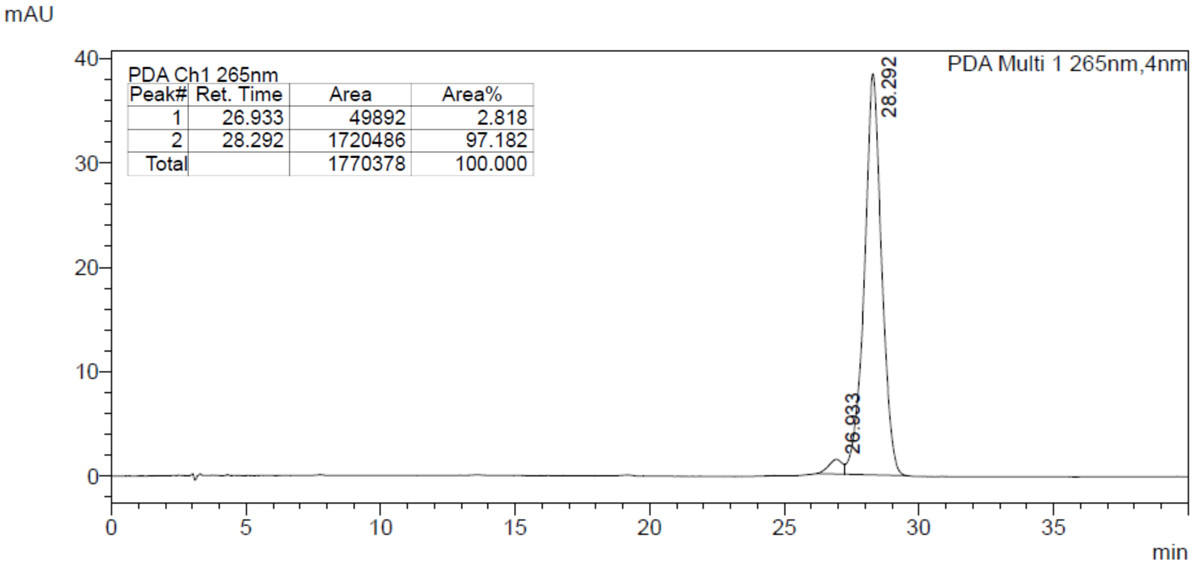

Figure S5 Chiral HPLC chromatogram of baclofen intermediate **17** (Table 5, entry 3)

<u>NMR spectra of demethylated cinchona squaramide (6)</u>

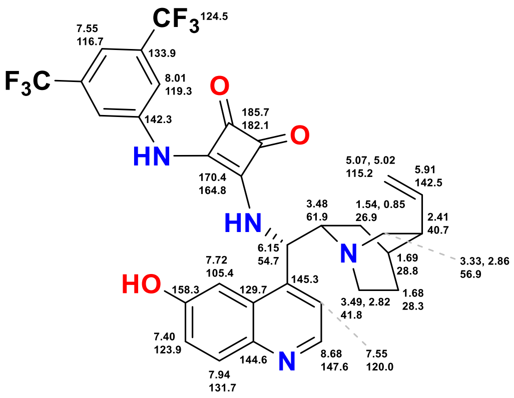

Figure S6 Molecule structure, ^{1}H and ^{13}C assignments of demethylated cinchona squaramide **6**

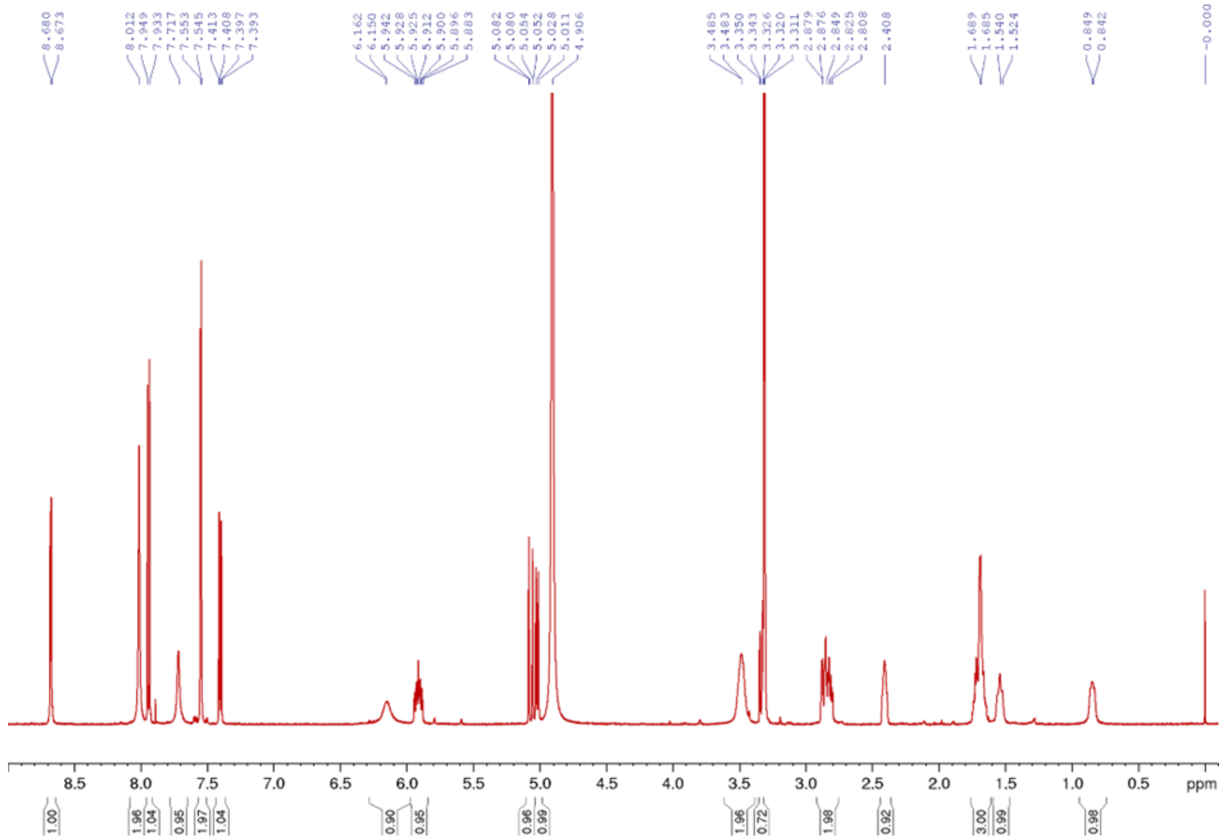

Figure S7 ^{1}H NMR spectrum of demethylated cinchona squaramide **6** (methanol-d_4)

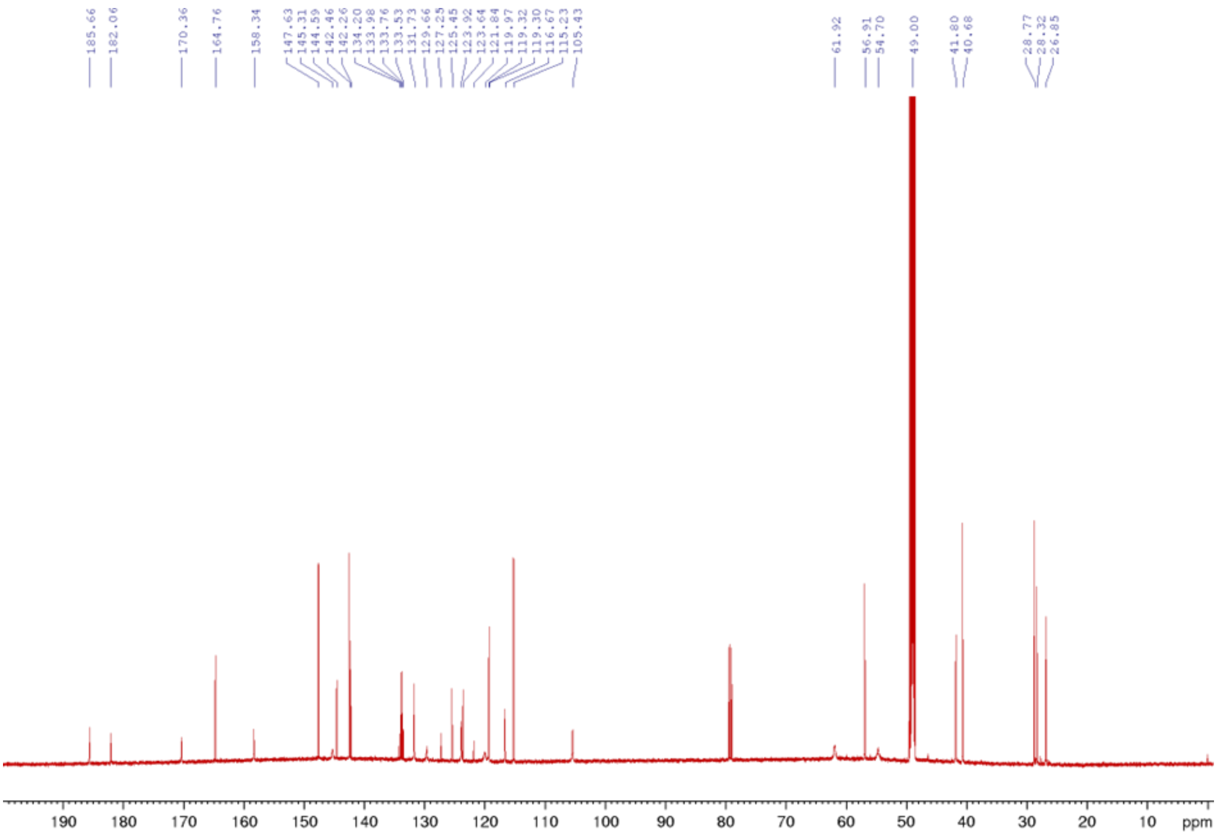

Figure S8 ^{13}C NMR spectrum of demethylated cinchona squaramide **6** (methanol-d_4)

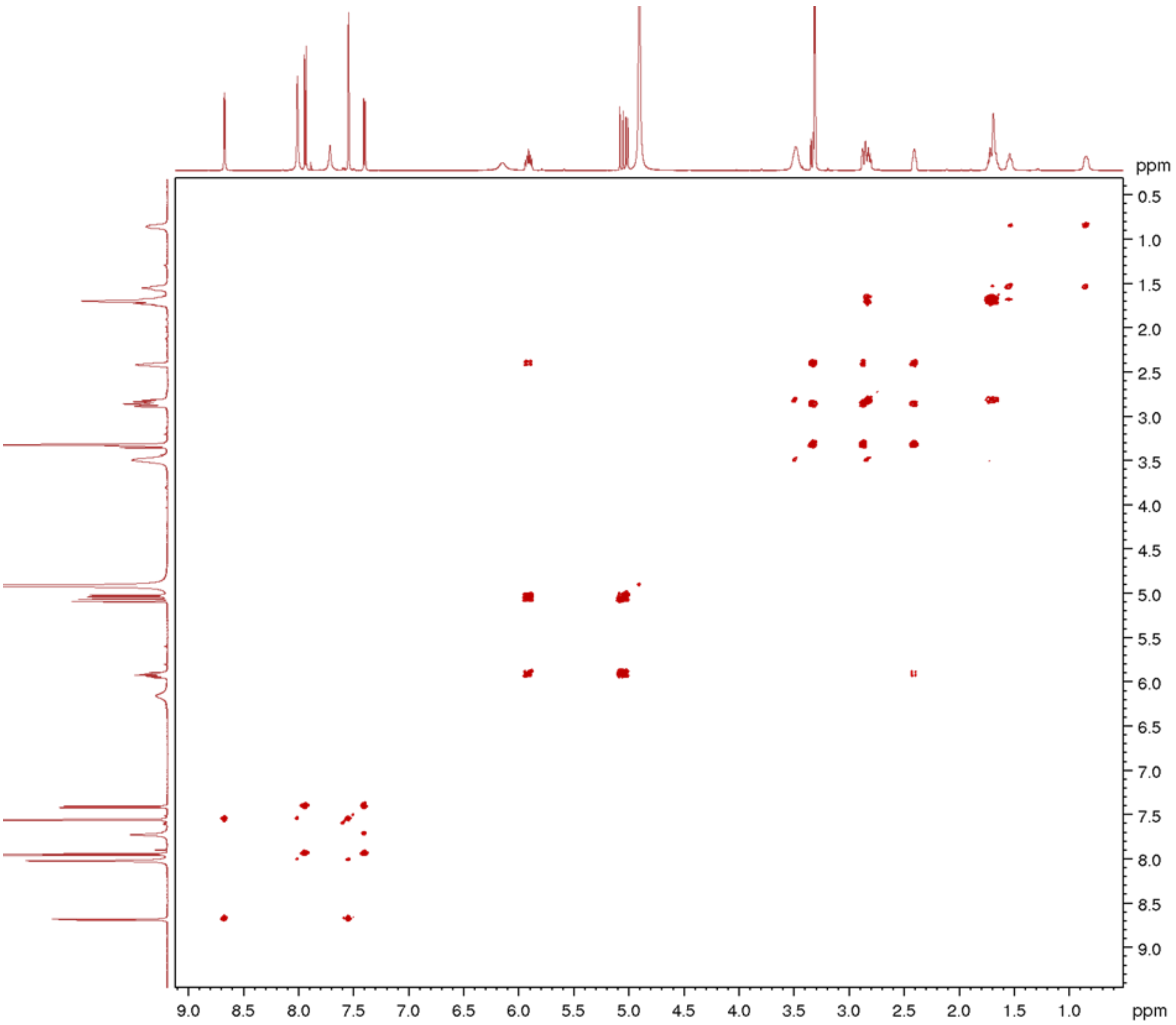

Figure S9 COSY spectrum of demethylated cinchona squaramide **6** (methanol-d_4)

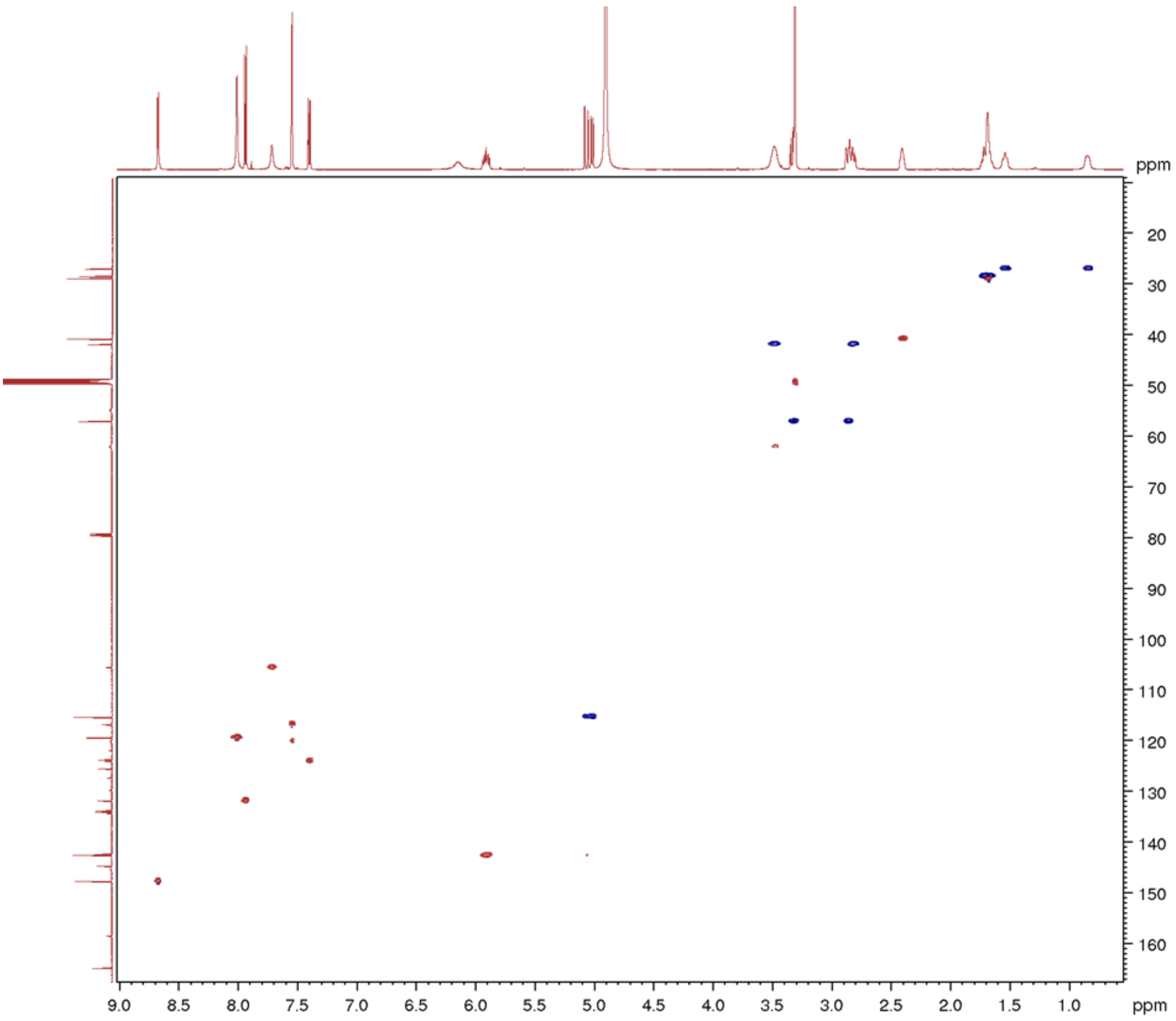

Figure S10 HSQC spectrum of demethylated cinchona squaramide **6** (methanol-d_4)

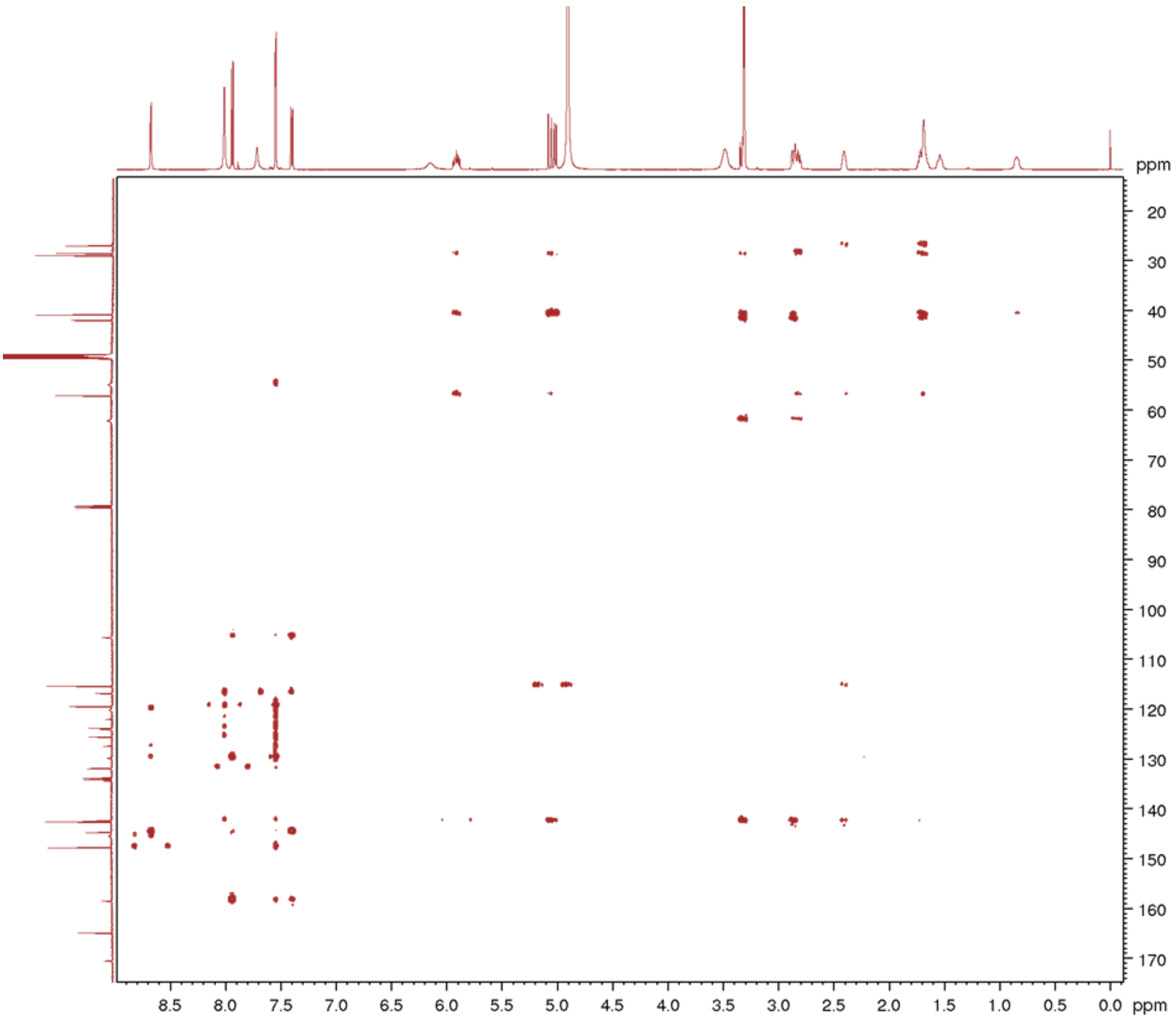

Figure S11 HMBC spectrum of demethylated cinchona squaramide **6** (methanol-d_4)

NMR spectra of cinchona squaramide organocatalyst with linker 7

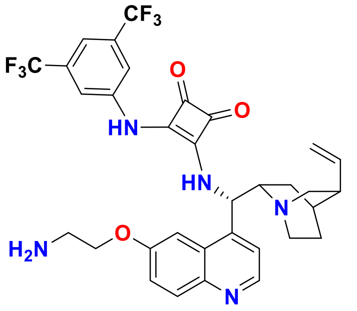

7

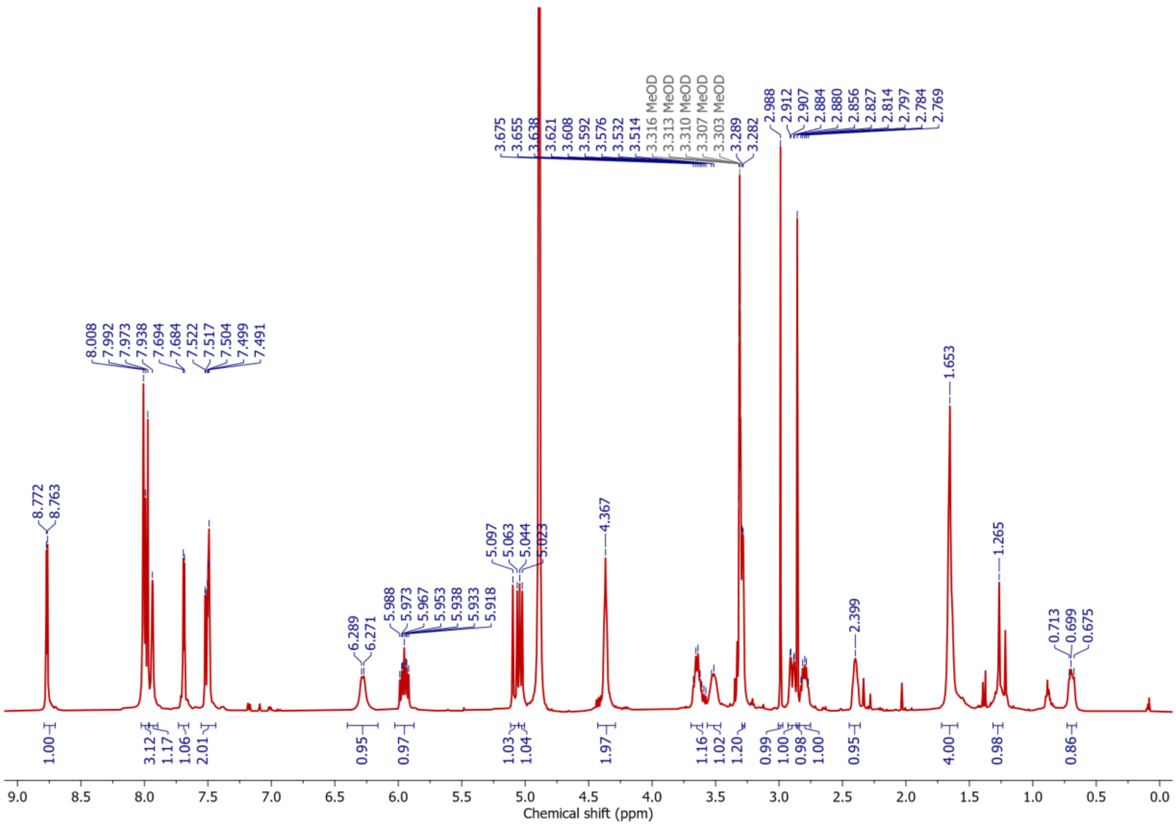

Figure S12 ^{1}H NMR spectrum of cinchona squaramide with linker **7** (methanol-d_4)

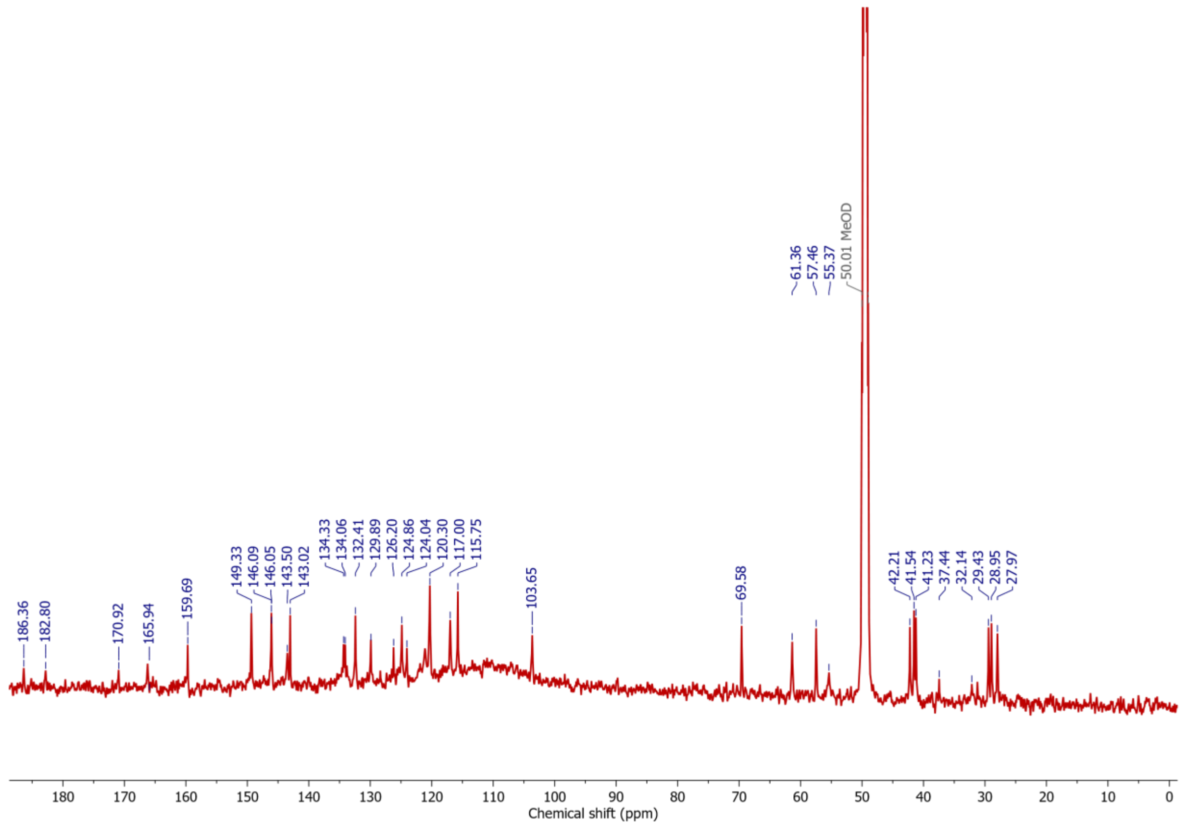

Figure S13 ^{13}C NMR spectrum of cinchona squaramide with linker **7** (methanol-d_4)

FT-IR spectrum of cinchona squaramide with linker 7

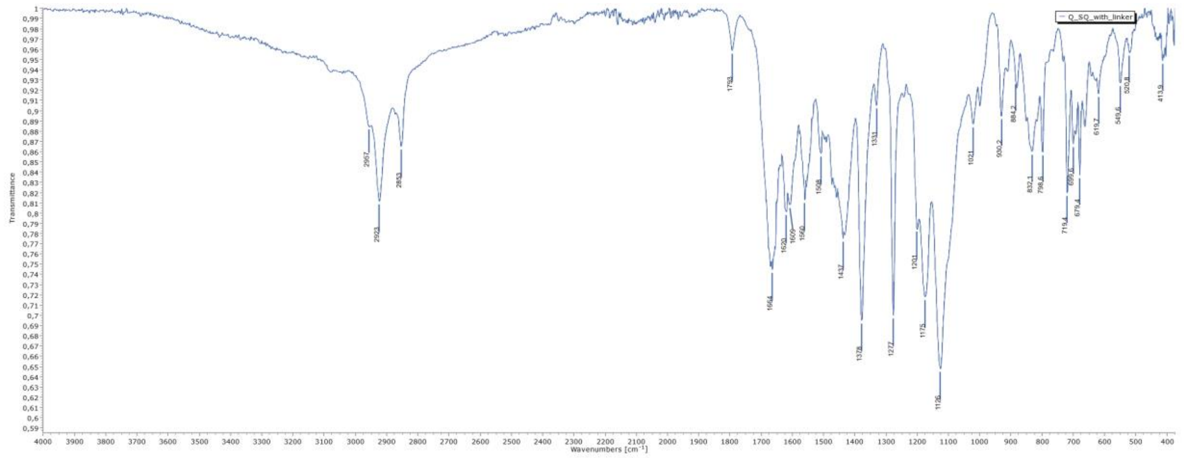

Figure S14 FT-IR spectrum of cinchona squaramide with linker **7**

NMR spectra of lipophilic organocatalyst 2

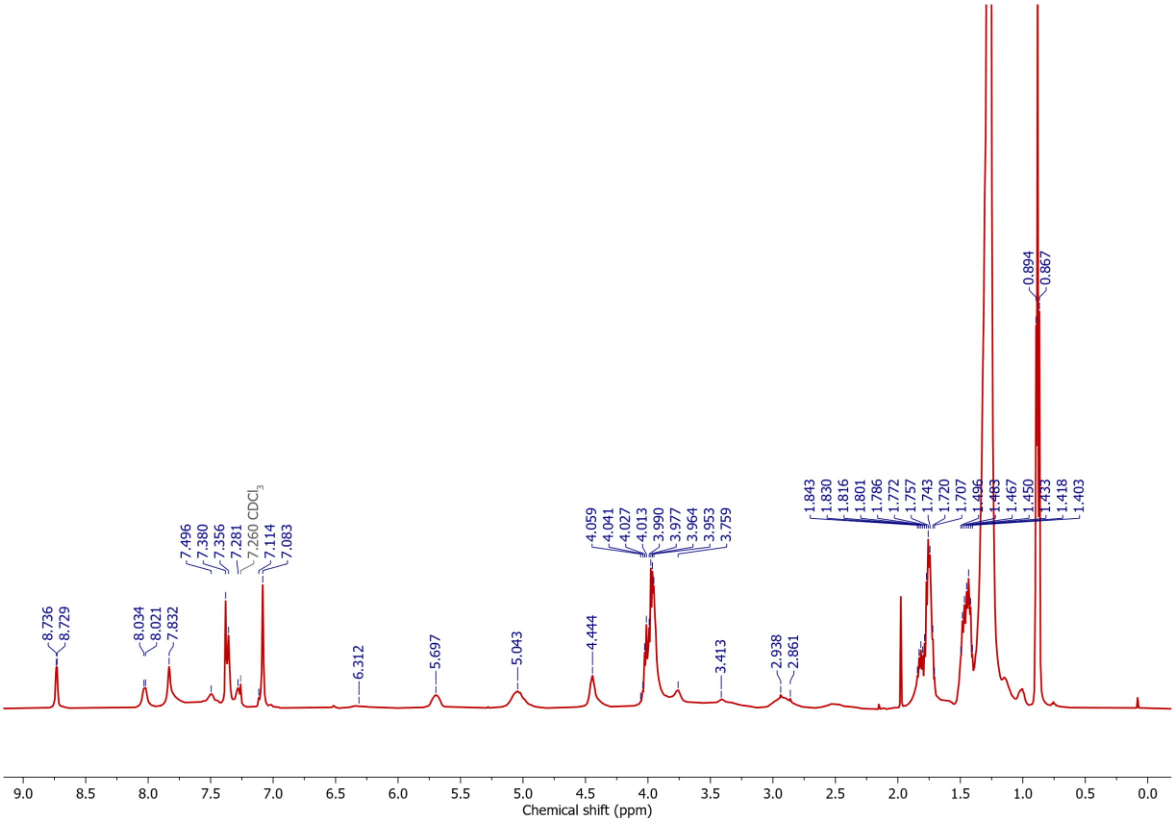

Figure S15 ^{1}H NMR spectrum of lipophilic organocatalyst **2** ($CDCl_3$)

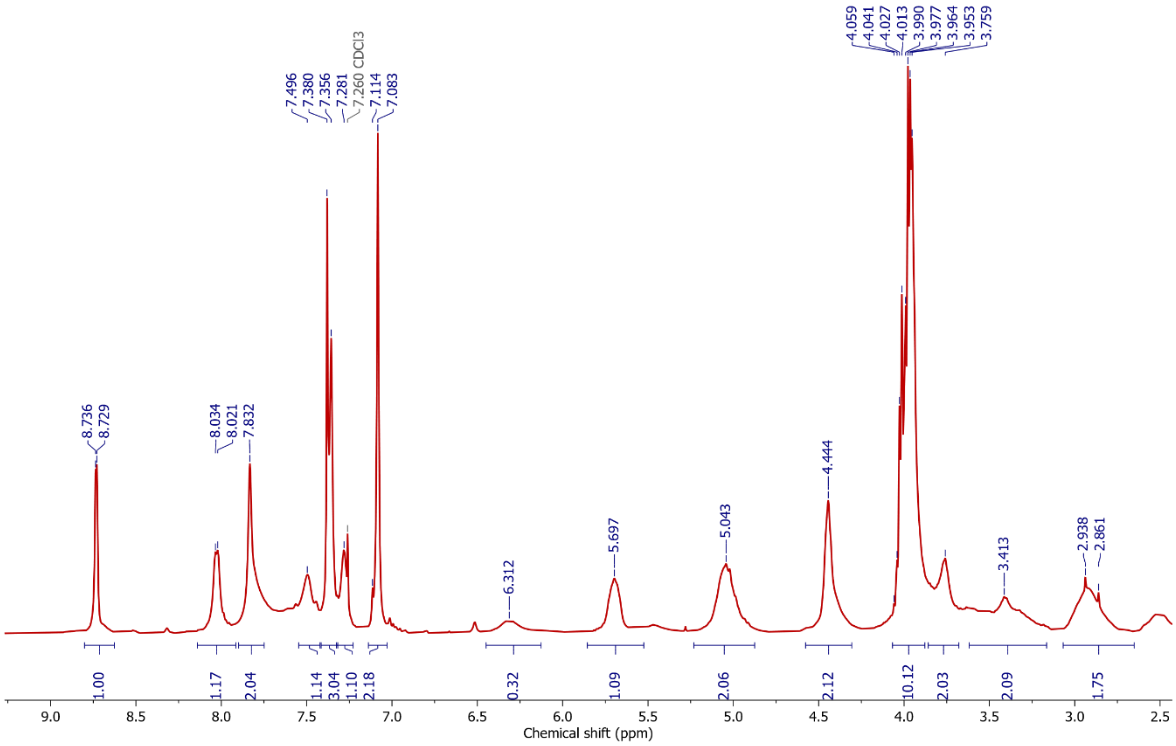

Figure S16 ^{1}H NMR spectrum of lipophilic organocatalyst **2** from 2.5 ppm to 9.0 ppm ($CDCl_3$)

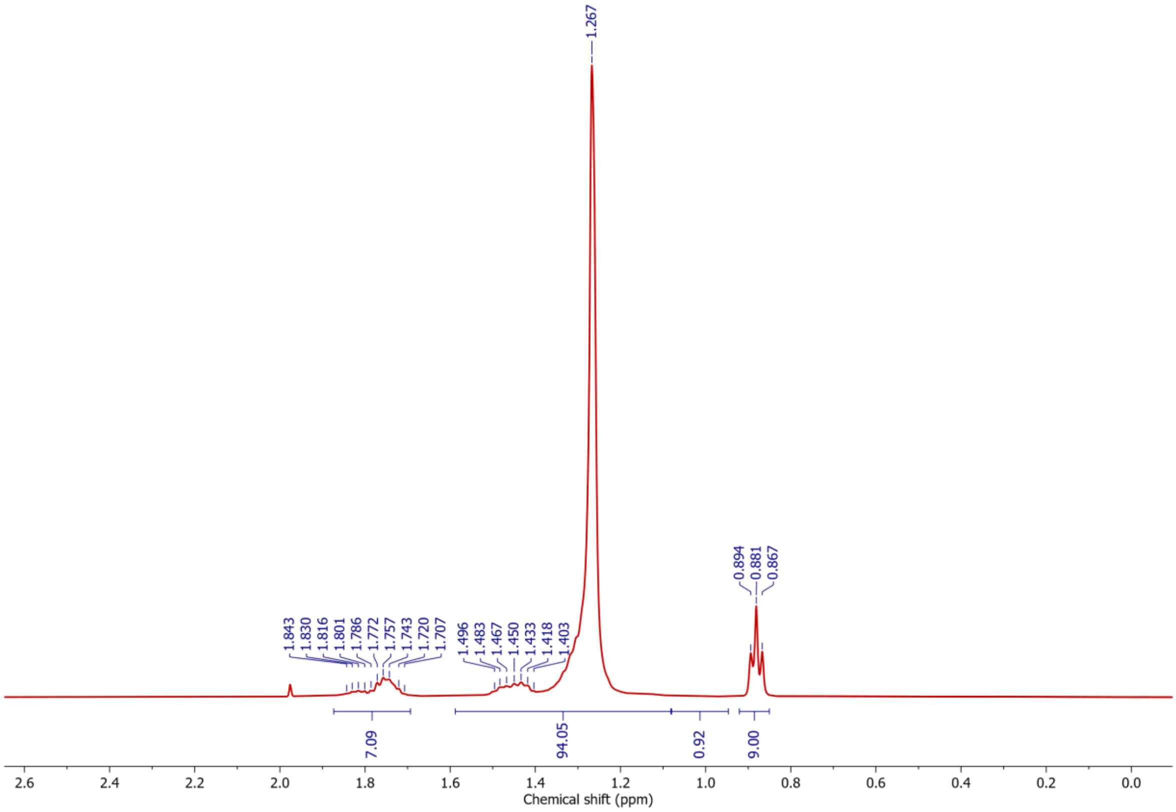

Figure S17 ^{1}H NMR spectrum of lipophilic organocatalyst **2** from 0 ppm to 2.6 ppm ($CDCl_3$)

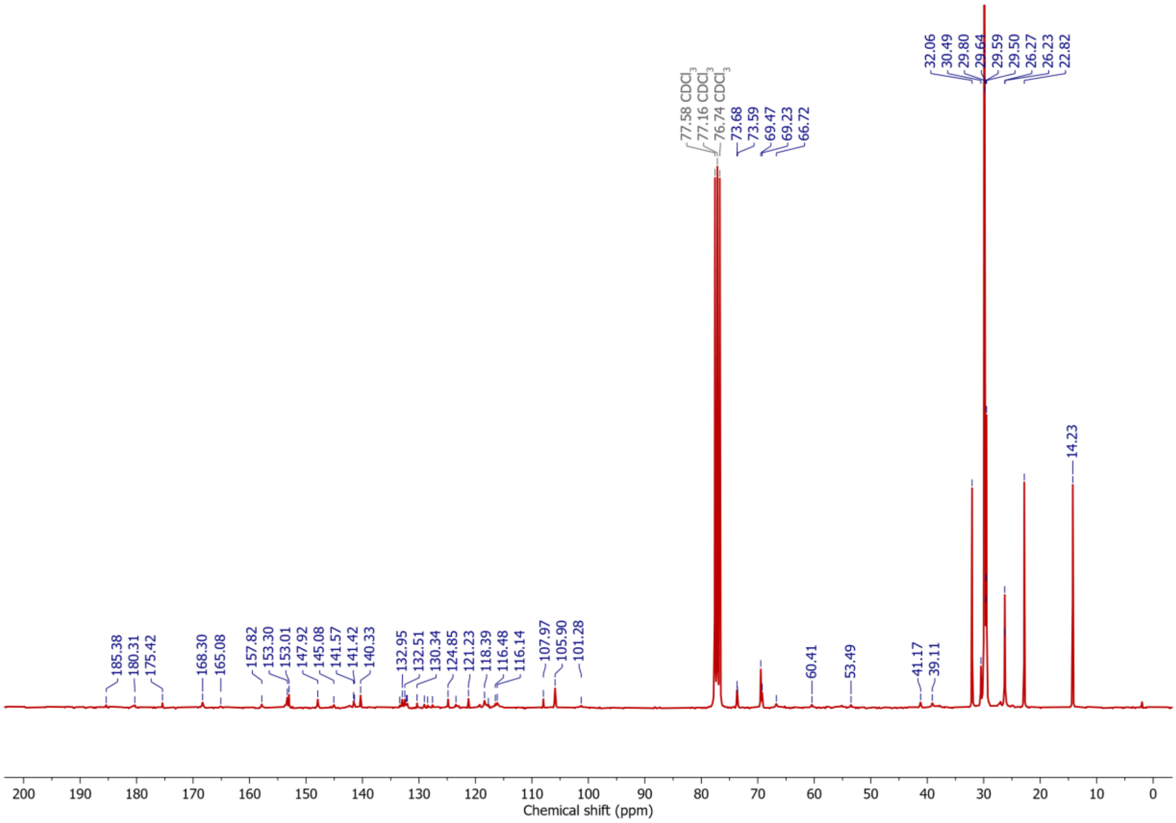

Figure S18 ^{13}C NMR spectrum of lipophilic organocatalyst **2** ($CDCl_3$)

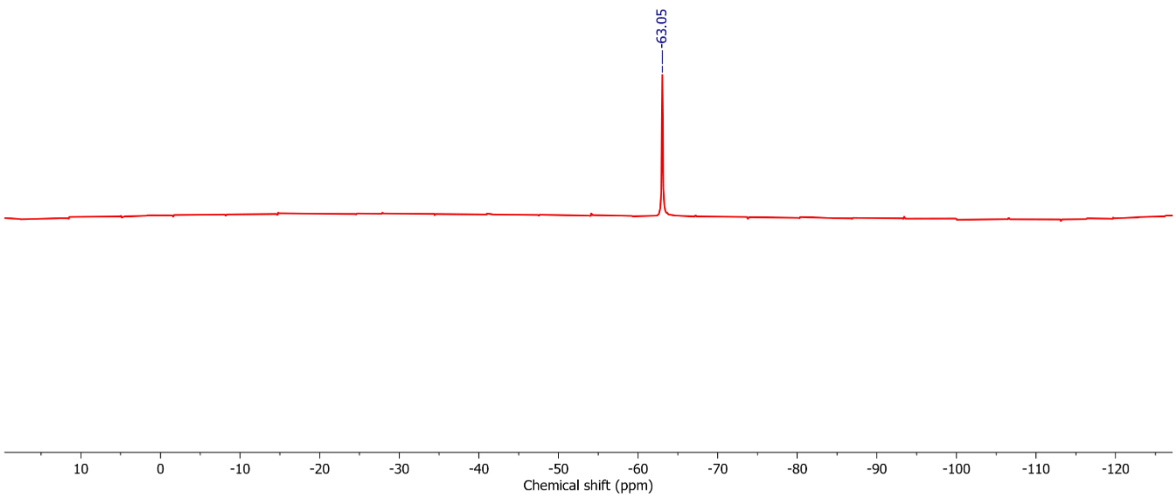

Figure S19 ^{19}F NMR spectrum of lipophilic organocatalyst **2** ($CDCl_3$)

FT-IR spectrum of lipophilic organocatalyst 2

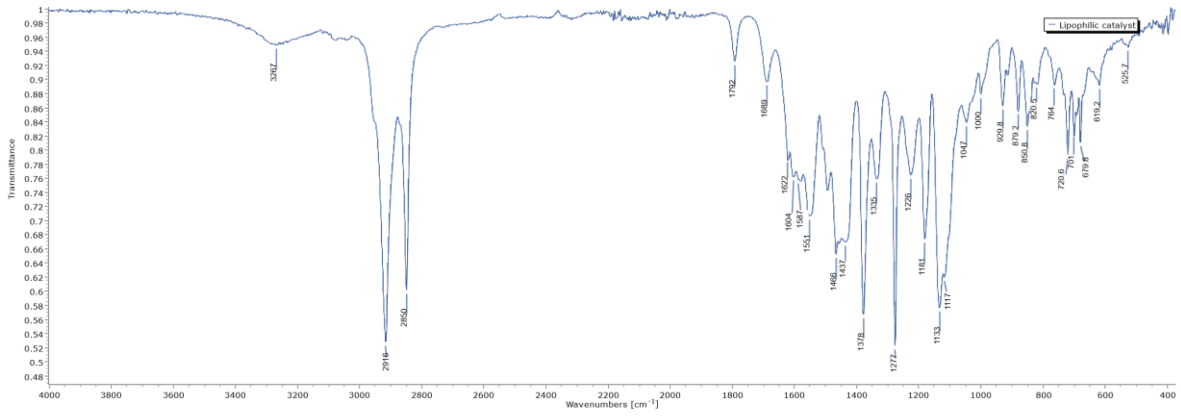

Figure S20 FT-IR spectrum of lipophilic organocatalyst (**2**)

NMR spectra of chiral baclofen intermediate 17

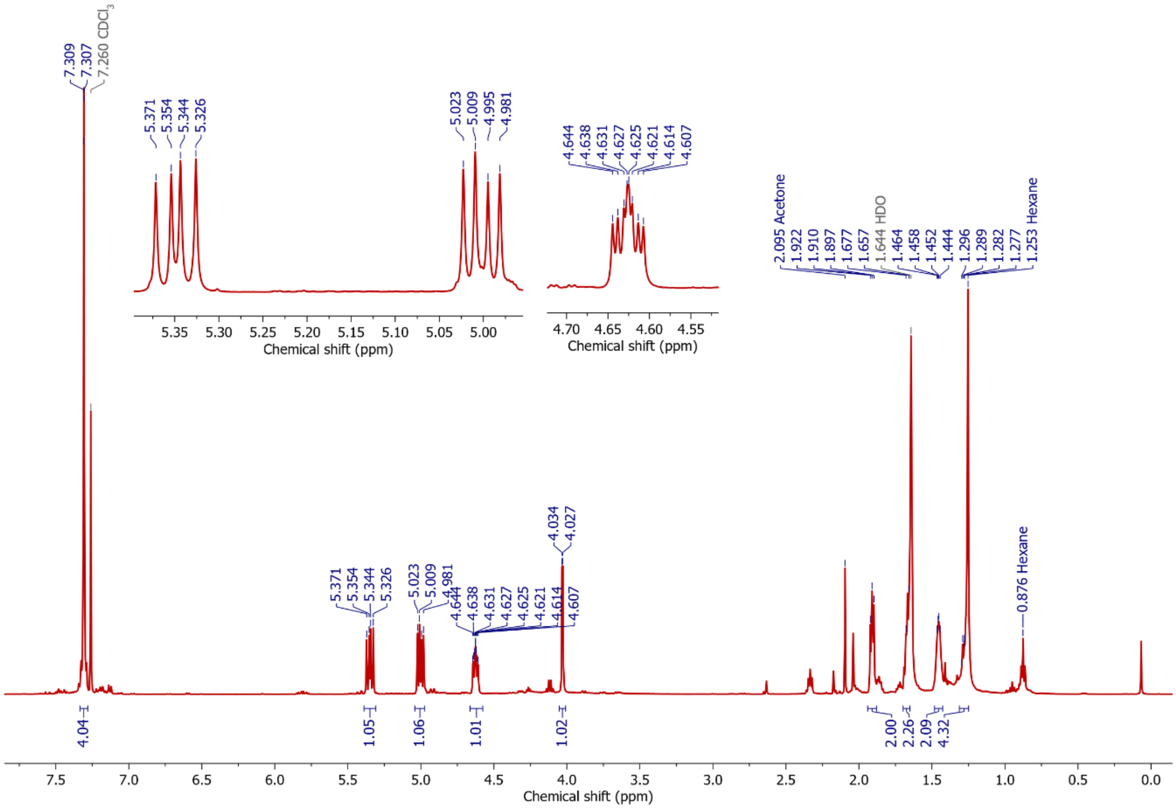

Figure S21 ^{1}H NMR spectrum of chiral baclofen intermediate **17** ($CDCl_3$)

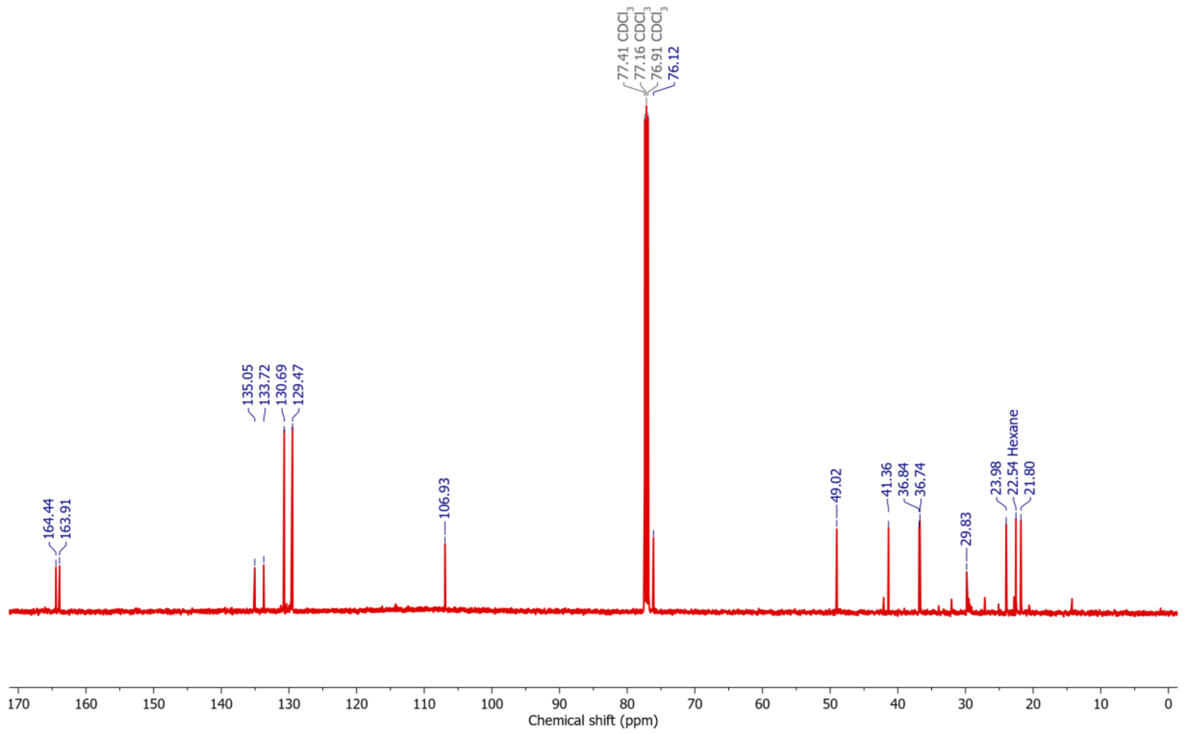

Figure S22 ^{13}C NMR spectrum of chiral baclofen intermediate **17** ($CDCl_3$)

FT-IR spectrum of chiral baclofen intermediate 17

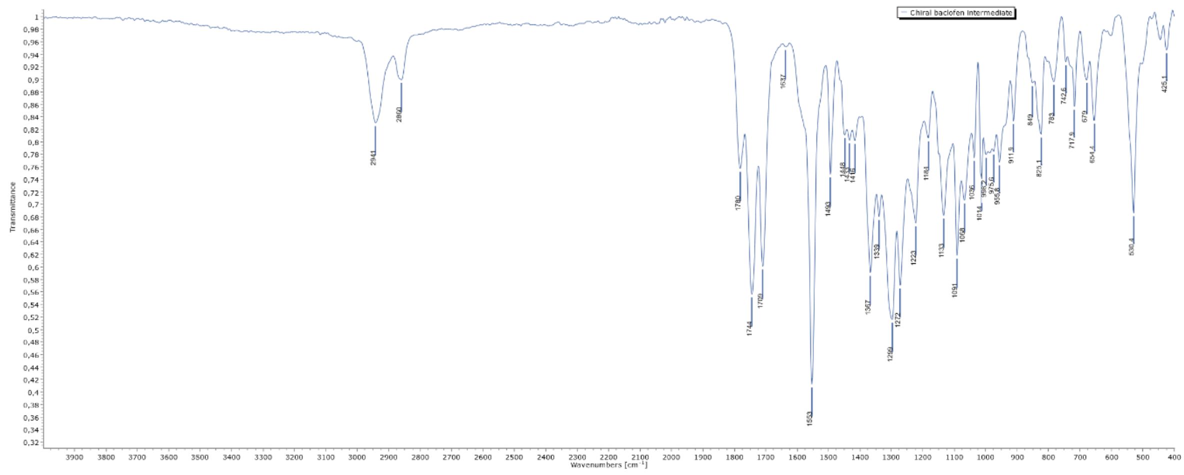

Figure S23 FT-IR spectrum of chiral baclofen intermediate **17**
